# Supplementary material for: Heterochromatic repeat clustering imposes a physical barrier on homologous recombination to prevent chromosomal translocations
Source: Mol Cell. 2022 Jun 2;82(11):2132–2147.e6. doi: 10.1016/j.molcel.2022.03.033 (PMC9616805; doi:10.1016/j.molcel.2022.03.033)
Supplement: Document S1. Figures S1–S6 and Tables S1–S3 [file mmc1.pdf]

Molecular Cell, Volume *82*

Supplemental information

Heterochromatic repeat clustering imposes

a physical barrier on homologous recombination

to prevent chromosomal translocations

Ioanna Mitrentsi, Jieqiong Lou, Adèle Kerjouan, John Verigos, Bernardo Reina-San-Martin, Elizabeth Hinde, and Evi Soutoglou

SUPPLEMENTARY Figure S1 (related to Figure 1)

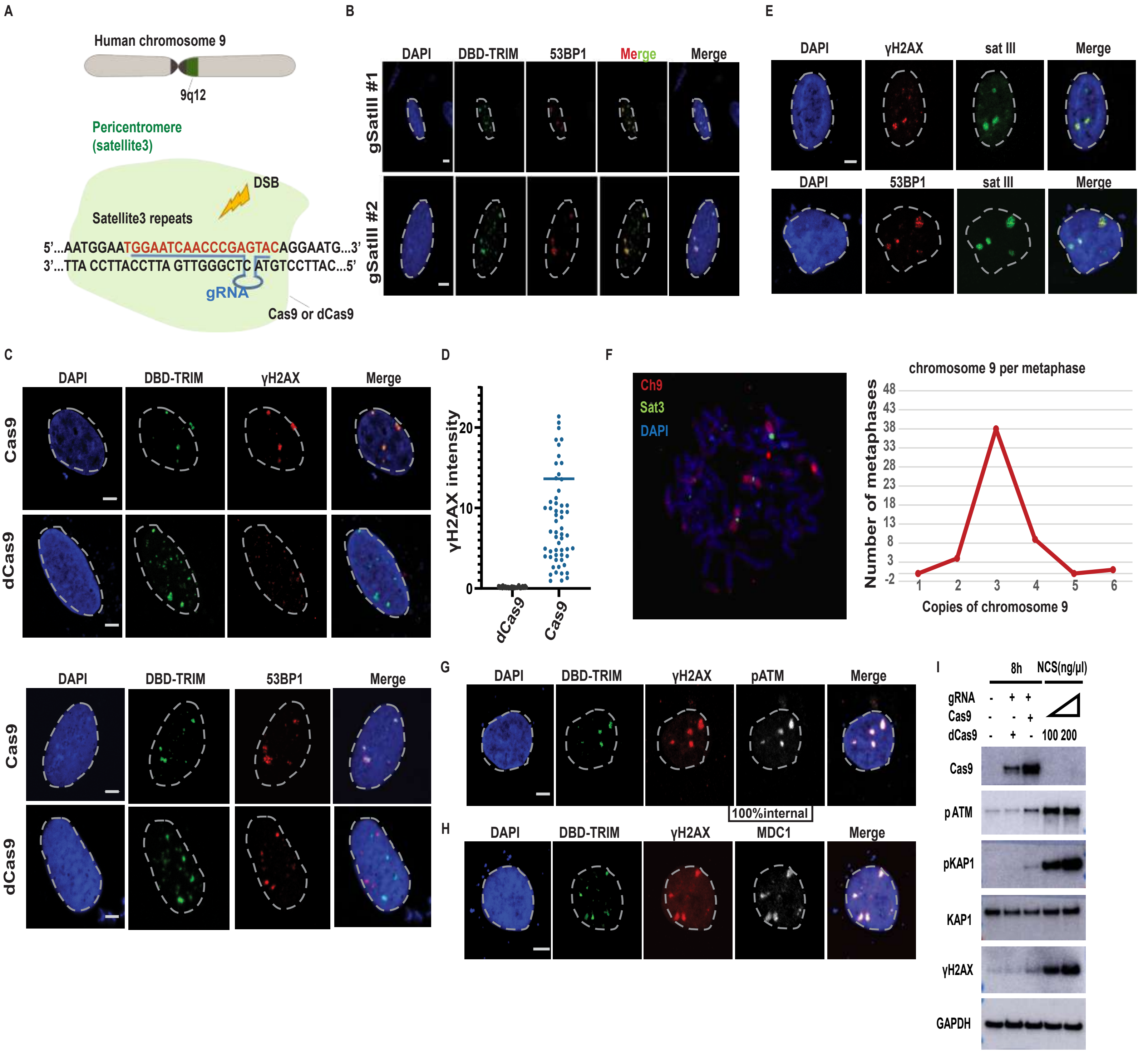

SUPPLEMENTARY Figure S2 (Related to Figure 1)

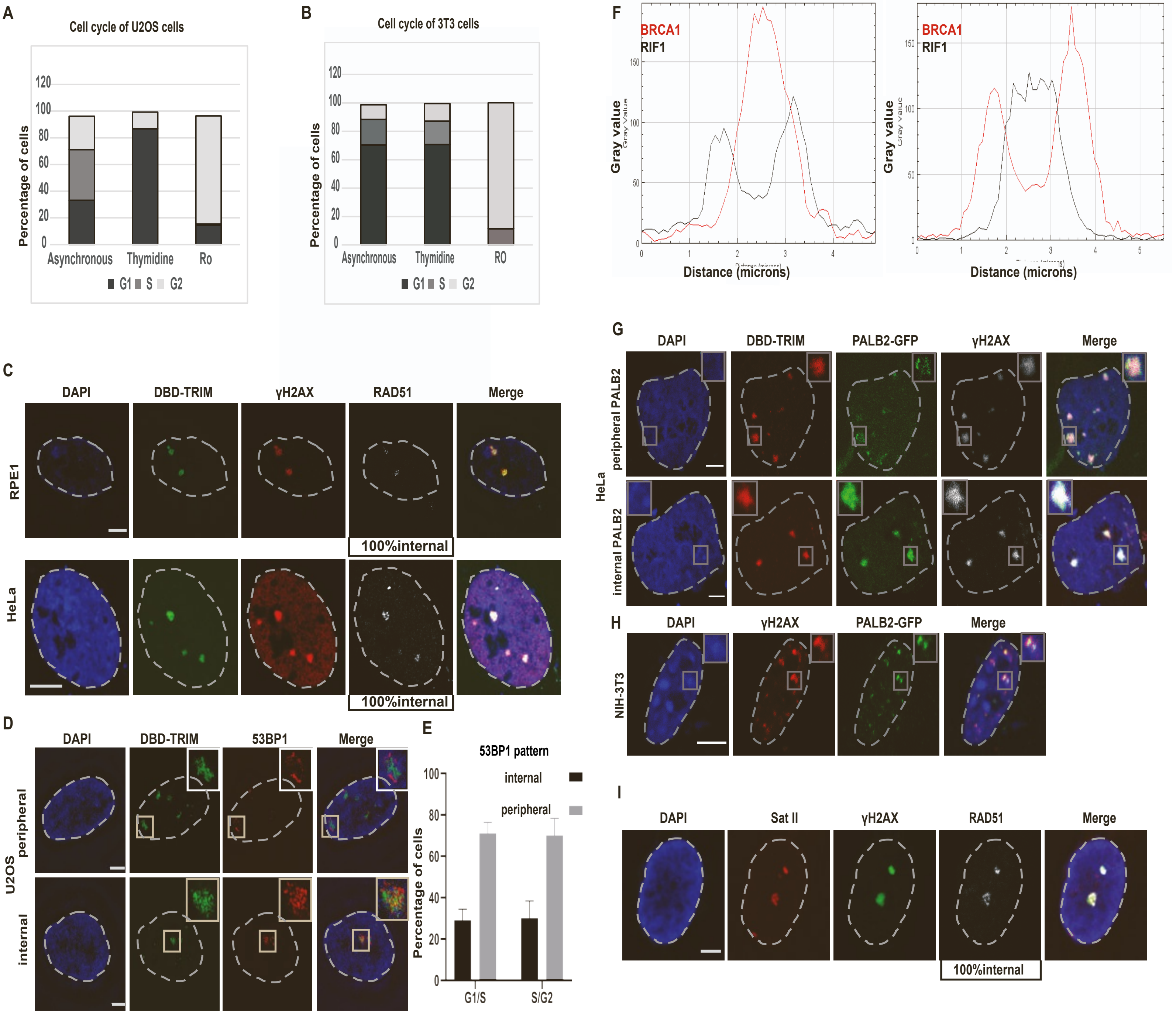

SUPPLEMENTARY Figure S3 (Related to Figure 2)

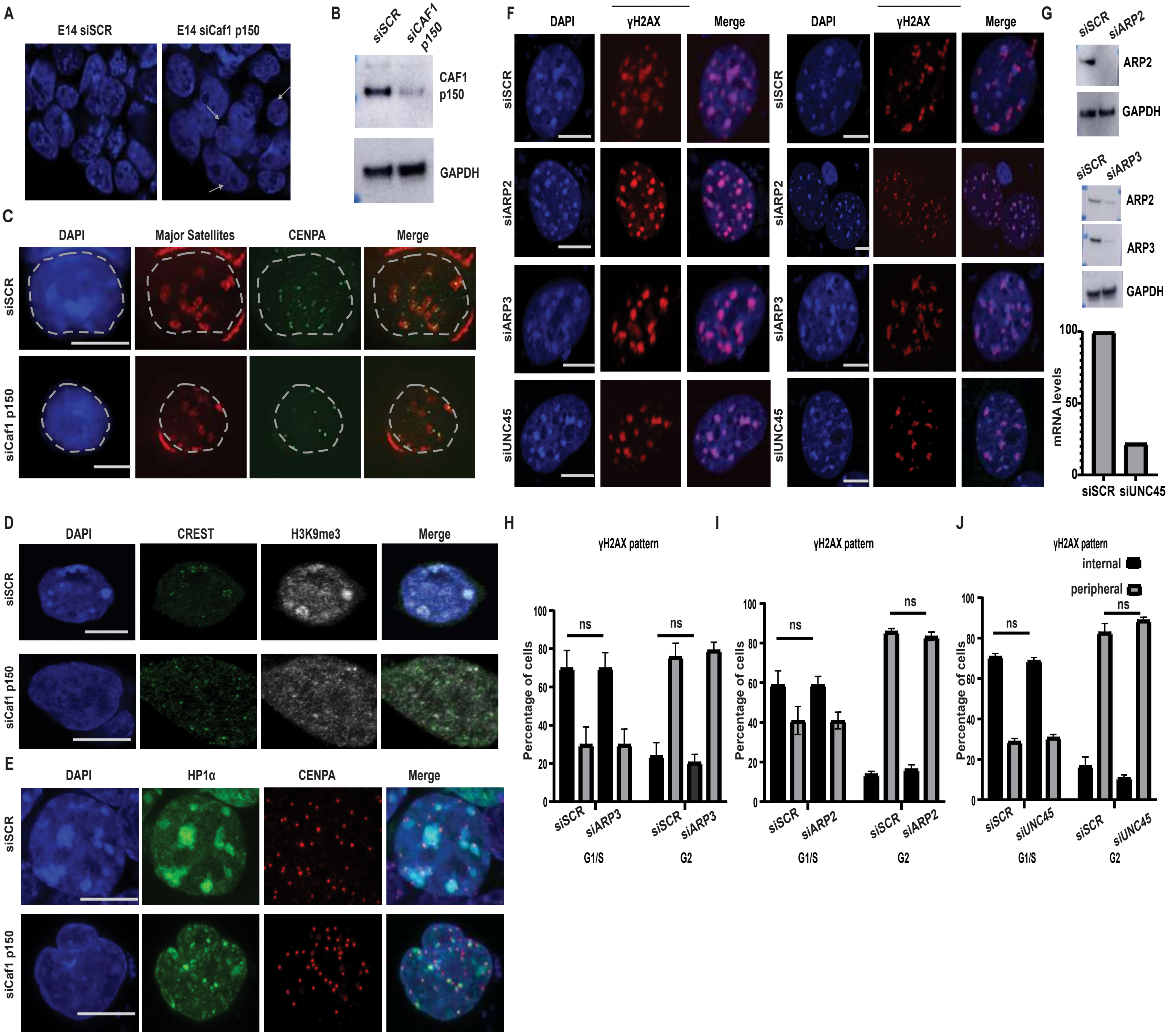

SUPPLEMENTARY Figure S4 (Related to Figure 4)

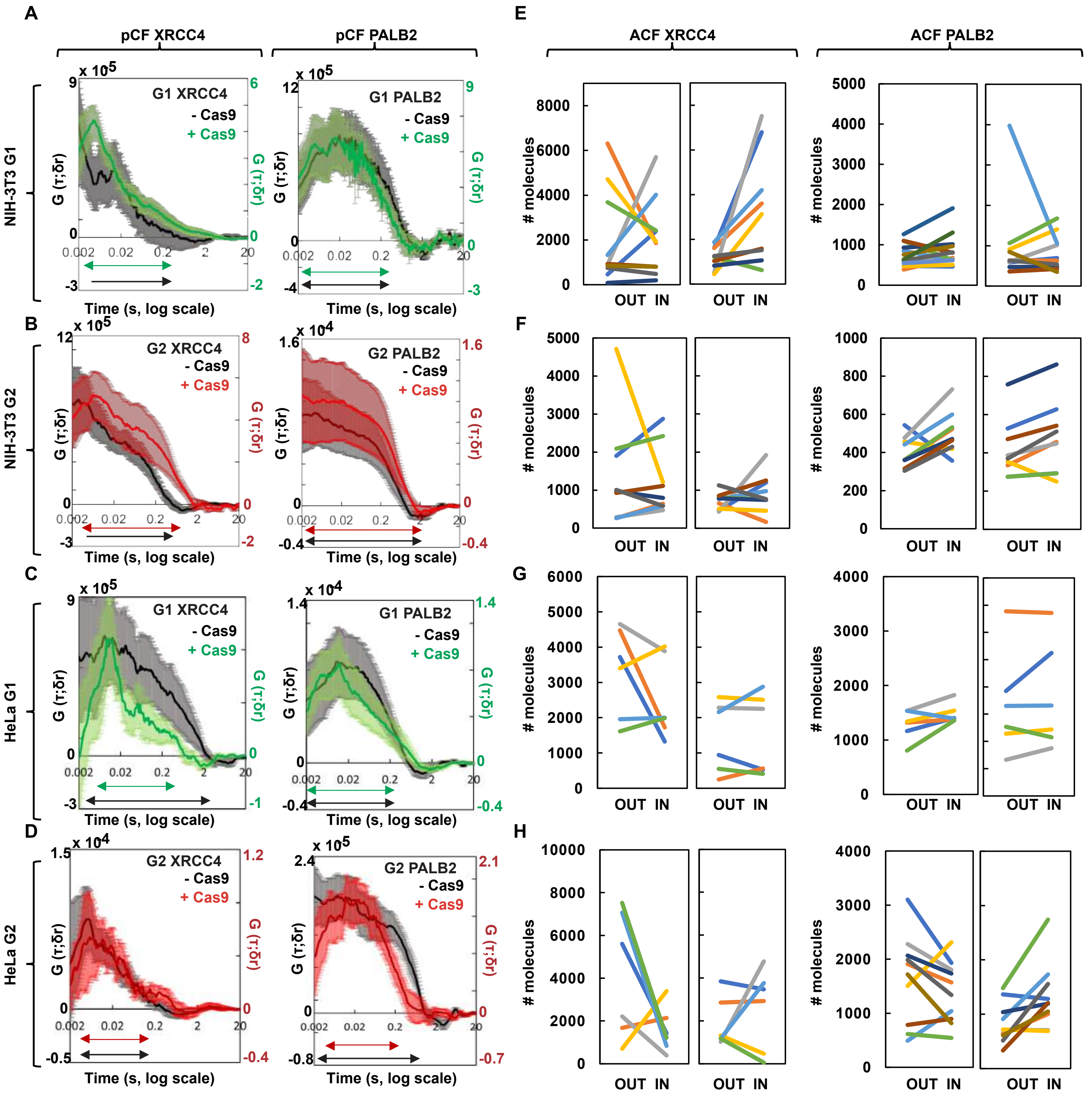

SUPPLEMENTARY Figure S5 (Related to Figure 5)

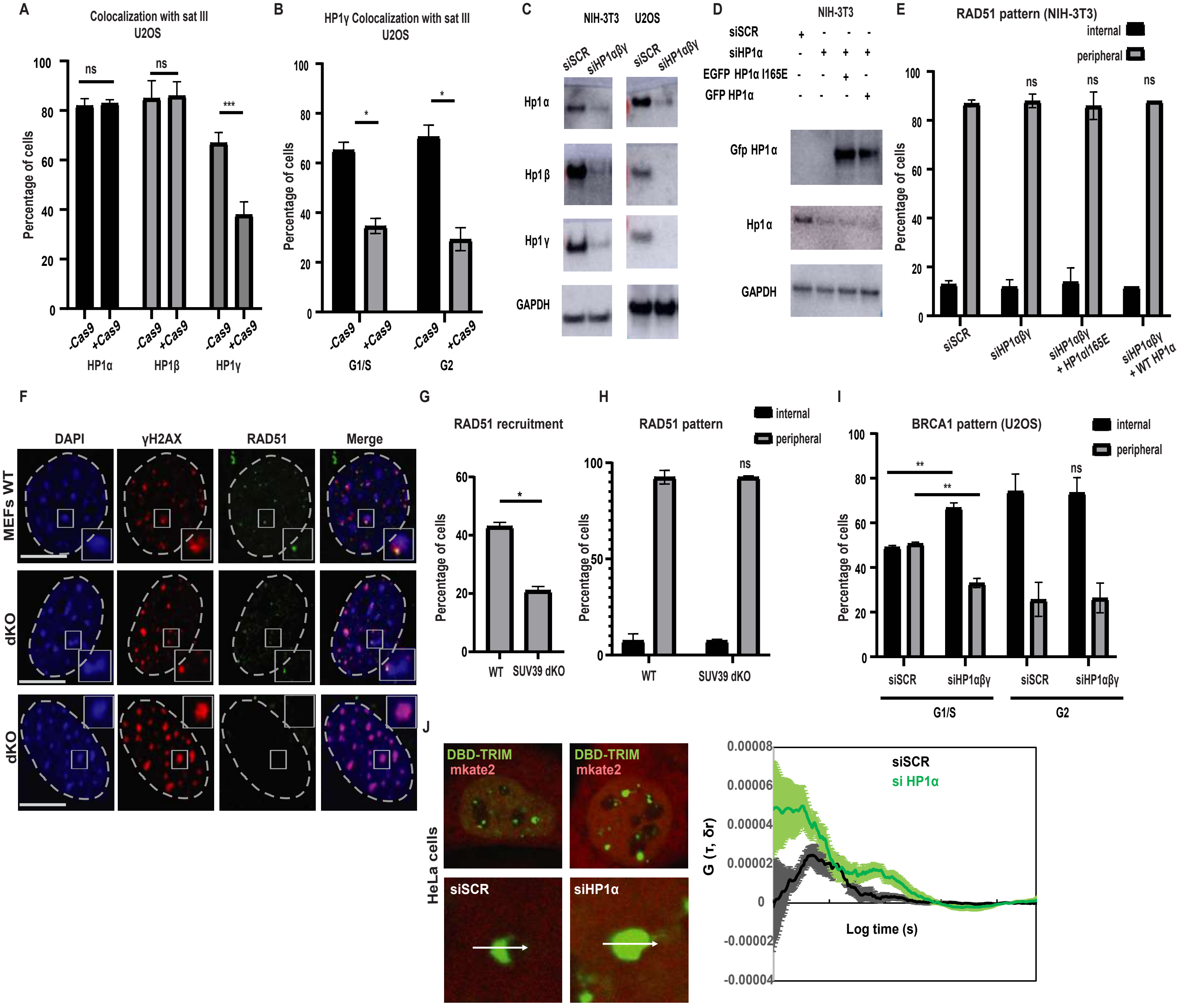

SUPPLEMENTARY Figure S6 (Related to Discussion)

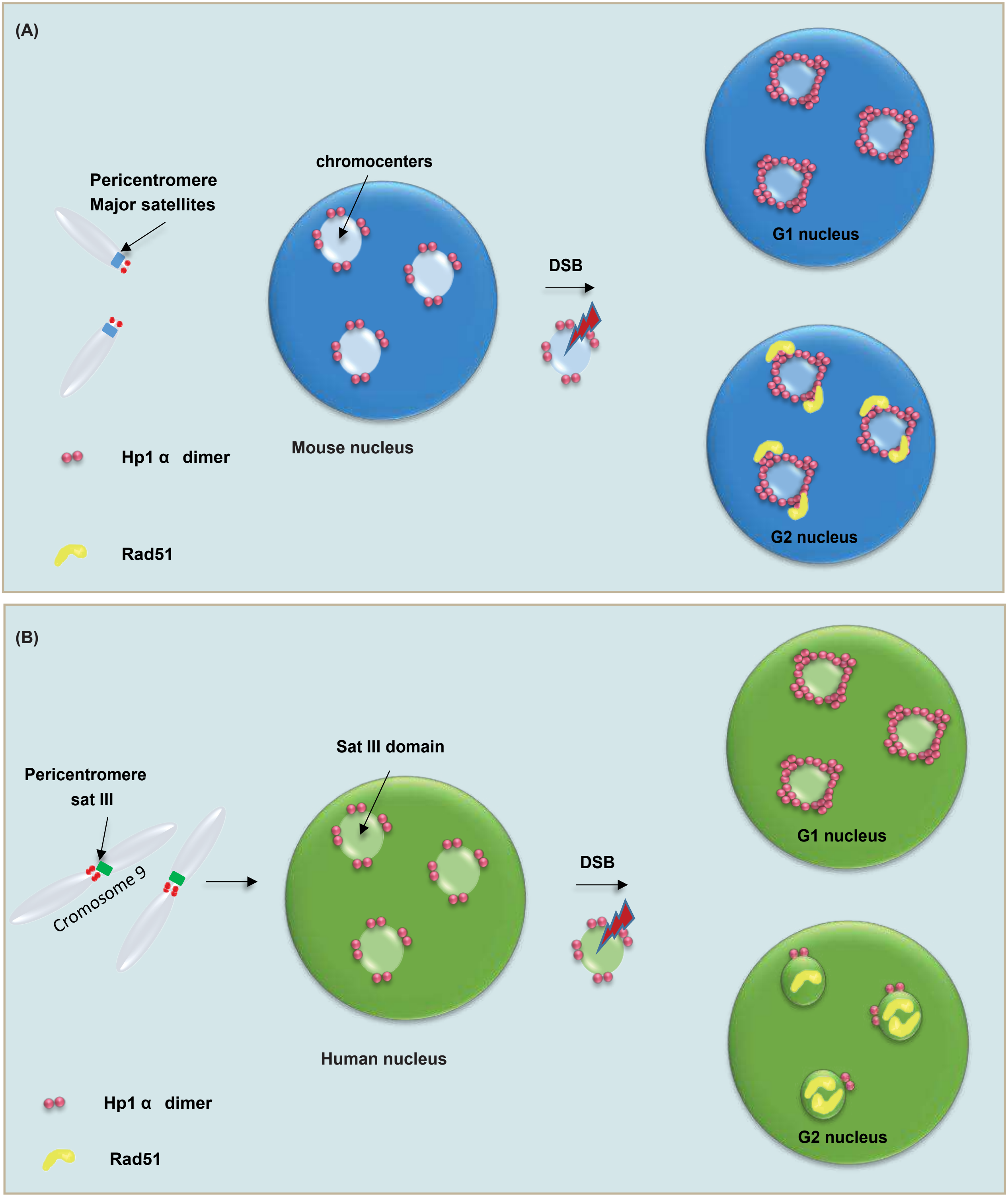

Supplementary Figure Legends

Figure S1 related to Figure1. Experimental system to induce Cas9 specific DSBs on human satIII pericentromeric regions (A) Schematic representation of the experimental system. Immunofluorescence (IF) confocal analysis of **(B)** cells co-expressing DBD-TRIM-EGFP+Cas9 + two different gRNAs specific for SatIII repeats stained with DAP1 and a 53BP1-specific antibody and **(C)** cells co-expressing DBD-TRIM-EGFP, a gRNA specific for SatIII repeats and either catalytically inactive dead Cas9 (dCas9) or Cas9 and stained with DAPI and antibodies specific for γH2AX and 53BP1. **(D)** Quantification of γH2AX intensity at sat III domains in cells co-expressing DBD-TRIM-EGFP, a gRNA specific for SatIII repeats and either dCas9 or Cas9 **(E)** IF combined with Fluorescence *in situ* Hybridization (ImmunoFISH) confocal analysis in U2OS cells expressing Cas9 + gRNA specific for the SatIII repeats stained with DAPI, a PNA-Sat III probe (green) and antibodies specific for γ-H2AX and 53BP1. **(F)** Representative confocal image of metaphase spreads after FISH with a PNA-SatIII probe (green) and chromosome paint (chromosome 9, red) and quantification of chromosome 9 copies per metaphase in U2OS cells. Data are the mean of 2 experiments (n= 50 cells). **(G-H)** IF confocal analysis of U2OS cells expressing DBD-TRIM-EGFP + Cas9 + gRNA specific for SatIII repeats stained with DAPI and antibodies specific for γ-H2AX, $pATM^{S1981}$ and MDC1. Images are representative of 3 experiments with n=50 cells. **(I)** Western blot analysis for Cas9, γ-H2AX, $pATM^{S1981}$, KAP1, $pKAP1^{S824}$ and GAPDH in protein extracts prepared from U2OS cells expressing Cas9 + gRNA specific for SatIII repeats or treated with NCS (100 ng/ml and 200 ng/ml).

Figure S2 related to Figure 1. Recruitment of HR and NHEJ factors at pericentromeric DSBs of different cell lines Cell cycle analysis by flow cytometry using propidium iodide and EdU in U2OS **(A)** and NIH-3T3 **(B)** cells treated or not with double thymidine (synchronization in G1/S) or RO-3306 (synchronization in S/G2. **(C)** IF and super resolution (upper panel) or confocal (lower panel) analysis of RPE1 (upper panel) or HeLa (lower panel) of cells expressing DBD-TRIM-EGFP+Cas9+gRNA targeting SatIII repeats stained with DAPI and antibodies specific for γ-H2AX and RAD51. **(D)** IF super resolution (3D-SIM) analysis of U2OS cells expressing DBD-TRIM-EGFP + Cas9 + gRNA targeting SatIII repeats stained with DAPI and a 53BP1-specific antibody. The upper panel represents a peripheral 53BP1 pattern and the lower internal. **(E)**Quantification of 53BP1 pattern after DSB induction in either G1/S or S/G2 synchronized U2OS cells. **(F)** Line scan analysis of BRCA1 and RIF1 spatial evolution at sat III domains on U2OS cells expressing Cas9+gRNA specific for sat III repeats. **(G)** IF confocal analysis of HeLa cells expressing DBD-TRIM-mCherry +PALB2-GFP+Cas9 + gRNA targeting SatIII repeats stained with DAPI and γH2AX specific antibody. Upper cell shows an internal PALB2 pattern whereas lower cell shows a peripheral PALB2 pattern. **(H)** IF confocal analysis of NIH-3T3 cells expressing PALB2-GFP, Cas9 + gRNA targeting major satellite repeats, stained with DAPI and γH2AX specific antibody. **(I)** ImmunoFISH confocal analysis of U2OS cells expressing Cas9 + gRNA targeting SatII repeats stained with DAPI, a SatII-specific probe, and antibodies specific for γH2AX and RAD51. Scale bars represent 5 µm. All images are representative of 3 experiments with n= 50 cells. Wherever a quantification is not provided, 100% of cells exert the phenotype.

Figure S3 related to Figure 2. Disruption of heterochromatic clustering on mouse ES cells and the role of actin/myosin mechanisms on DSB relocation (A) Confocal analysis of WT E14 cells or E14 cells depleted for Caf1 p150 (siCAF1p150; right panel) or with a non-targeting control (siSCR; left panel) stained with DAPI. The arrows indicate cells with the phenotype of disrupted clustering **(B)** Western blot analysis of CAF1 levels at WT E14 cells and E14 cells depleted of Caf1 p150. **(C)** ImmunoFISH confocal analysis of WT E14 cells (upper panel) or E14 cells depleted for Caf1 p150 (lower panel) cells expressing Cas9 + gRNA targeting major satellite repeats stained with DAPI, a major satellite-specific probe and a CENP-A specific antibody. Images are representative of 3 experiments with n = 50 cells. IF confocal analysis of WT E14 cells or E14 cells depleted for Caf1 p150, stained with DAPI and antibodies specific for CREST and H3K9me3 **(D)** or HP1α and CENPA **(E). (F)** IF confocal analysis of NIH-3T3 cells expressing Cas9 + gRNA targeting major satellites, depleted for ARP2 (siARP2), ARP3 (siARP3), UNC45 (siUNC45) or a non-targeting control (siSCR) in different stages of the cell cycle and stained with DAPI and an antibody specific for γH2AX. **(G)** siRNA efficiencies of siARP2, siARP3 (by western blot analysis) and siUNC45 (by RT-qPCR). **(H-J)** Quantification of the γH2AX pattern at chromocenters of NIH-3T3 cells expressing Cas9 + gRNA specific for the major satellite repeats, either depleted of ARP3 (siARP3) **(H)**, ARP2 (siARP2) **(I)** , or UNC45(siUNC45) **(J)** or with a non-targeting control (siSCR) in G1 or G2 phases of the cell cycle. Scale bars represent 5 μm. Data are the mean ± SD of 3 experiments with n=50 cells. For all graphs, statistical significance was determined by t-test (*p<0,05, **p<0,01, ***p<0,001).

Figure S4 related to Figure 4 pCF and ACF analysis of PALB2 and XRCC4 access on mouse versus human heterochromatin, throughout the cell cycle pCF analysis of the access of PALB2-GFP or mCherry-XRCC4 into heterochromatic DSBs and ACF analysis of the fraction of each DNA repair

factor present and bound within the indicated nuclear structures. **(A)-(B)** pCF6-8 (δr = 6-8 pixels) analysis of XRCC4 (left) versus PALB2 (right) access into NIH-3T3 in G1 **(A)** and G2 **(B)** heterochromatin in the absence (grey) versus presence (G1-green, G2-red) of DSBs (N=6-12 measurements, n>= 5cells, 2 biological replicates) and ACF (δr=0 pixels). **(C)-(D)** pCF6-8 (δr = 6-8 pixels) analysis of XRCC4 (left) versus PALB2 (right) access into HeLa in G1 **(C)** and G2 **(D)** heterochromatin in the absence (grey) versus presence (G1-green, G2-red) of DSBs (N=8-10 measurements, n>=5 cells, 2 biological replicates). **(E)-(F)** ACF analysis (δr = 0 pixels) of XRCC4 (left) versus PALB2 (right) mobility inside NIH-3T3 in G1 **(E)** and G2 **(F)** heterochromatin enables extraction of the number of moving molecules outside versus inside this nuclear structure, which underpins calculation of the ratios presented in Fig. 4J and L (N=6-12 measurements, n>= 5cells, 2 biological replicates). **(G)-(H)** ACF analysis (δr = 0 pixels) of XRCC4 (left) versus PALB2 (right) mobility inside HeLa in G1 **(G)** and G2 **(H)** heterochromatin enables extraction of the number of moving molecules outside versus inside this nuclear structure, which underpins calculation of the ratios presented in Fig. 4P and R). (N=8-10 measurements, n=5 cells, 2 biological replicates).

Figure S5 related to Figure 5. Localization of HR factors at heterochromatic DSBs in the absence of HP1s (A) Quantification of HP1α, Hp1β or HP1γ colocalization with SatIII domains in U2OS cells expressing a gRNA specific for SatIII repeats with or without Cas9-specific DSB induction. **(B)** Quantification of HP1γ colocalization with SatIII repeats in U2OS cells expressing a gRNA specific for SatIII repeats with or without Cas9-specific DSB induction in cells in different stages of the cell cycle. **(C)** Efficiency of siHP1αβγ siRNAs in U2OS and NIH-3T3 cells revealed by Western blot analysis. **(D)** Western blot analysis of the endogenous HP1α and GFP-HP1α or EGFP-HP1α^{I165E} levels in WT NIH-3T3 cells or NIH-3T3 cells depleted of Hp1αβγ, before and after expression of

GFP-HP1α or EGFP-HP1α^{I165E}. **(E)** Quantification of RAD51 pattern at chromocenters on G2-synchronized NIH-3T3 cells expressing Cas9 + gRNA targeting major satellite repeats, depleted of Hp1αβγ and complemented with EGFP-HP1α^{I165E} or GFP-HP1α. **(F)** IF confocal analysis of WT MEFs (upper panel) or SUV3/9 dKO MEFs (middle and lower panels) expressing Cas9 + gRNA targeting major satellite repeats stained with DAPI and antibodies specific for γ-H2AX and RAD51. The lower panel represents a representative image of a cell where RAD51 is not recruited. Quantification of RAD51 recruitment **(G)** and pattern **(H)** in WT MEFs and SUV3/9 dKO MEFs expressing Cas9 + gRNA targeting major satellite repeats. Images are representative of 3 experiments with n=50 cells. **(I)** Quantification of BRCA1 pattern in U2OS cells expressing Cas9 + gRNA targeting SatIII repeats, depleted of HP1αβγ (siHP1αβγ) or transfected with a non-targeting control cells (siSCR) in G1/S (left) and S/G2 (right) phase of the cell cycle. **(J)** Two-colour confocal image of wt HeLa cells or HeLa cells depleted of HP1α expressing mKate2 and DBD-TRIM-EGFP, where a line scan (white arrow) in the region of interest was selected for pCF analysis, enlarged (bottom row). pCF6-8 analysis of mKate2 access into HeLa heterochromatin foci before (grey) versus after (green) HP1α depletion (N = 10 measurements across N>=5 cells and 1 biological replicate). Scale bars represent 5 µm. Data are the mean ± SD of 3 experiments with n = 50 cells. Statistical significance was determined by t-test (*p<0,05, **p<0,01, ***p<0,001).

Figure S6 related to Discussion. Model of the spatial activation of DNA repair pathways in DSBs occurring in mouse and human heterochromatin. (A) In mouse cells, chromosomes are acrocentric and cluster through their pericentromeric repeats to form chromocenters. In S/G2, DSBs induced at the chromocenters relocate to the periphery of the domain to be repaired by HR and recruit Rad51.

At the same time, HP1 dimers are formed at the periphery of the domain, leading to reduced accessibility to HR factors. **(B)** In human cells, sat III repeats located on chromosome 9 do not exert the same degree of clustering. When DSBs are induced at satIII domains, the breaks remain positionally stable, the Hp1 dimers are reduced and the domain becomes accessible to HR factors, such as Rad51.

Table S1 (related to STAR METHODS), Probes for FISH

Probes for FISH		
**Probe**	**Company (reference)**	**Sequence**
TelG-Alexa488 PNA	Panagene (F1008)	G-rich probe (repeats of TTAGGG)
Sat III PNA - Alexa488	Panagene	Biotin-O-TTCCATTCCATTCCATTCCA
Sat II	Sigma	Biotin-TCGAGTCCATTCGATGAT-Biotin
Human chromosome 9	Applied Spectral Imaging (FPRPR0166)	

Table S2 (Related to STAR METHODS), Oligos

**Target**	**Forward**	**Reverse**
GAPDH (RT-qPCR)	AACTTTGGCATTGTGGAAGG	ACACATTGGGGGTAGGAACA
UNC45 (RT-qPCR)	TTTCCTGGCGAGCGGATCTA	TCAATCATGGGCACAGCCTT
Caf1 p150 siRNA oligos	AAGGAGAAGGCGGAGAAGCAG[dT][dT]	CUGGUUCUCCGCCUUCUCCUU[dT][dT] (antisense)

Table S3 (related to STAR METHODS), gRNA sequences

Guide RNAs used	***Sequence/Source***
g347 (sat III #1)	AATGGAATCAACACGAG
g349 (sat III #2)	TGGAATCAACCCGAGTAC
Ma-sat#3	Tsouroula et al. 2016
g501 (sat II)	CCAGTGTGAGCATCATCGAA
