# Supplementary material for: Heterochromatic repeat clustering imposes a physical barrier on homologous recombination to prevent chromosomal translocations
Source: Mol Cell. 2022 Jun 2;82(11):2132–2147.e6. doi: 10.1016/j.molcel.2022.03.033 (PMC9616805; doi:10.1016/j.molcel.2022.03.033)
Supplement: Document S2. Article plus supplemental information [file mmc2.pdf]

Molecular Cell

Article

Heterochromatic repeat clustering imposes a physical barrier on homologous recombination to prevent chromosomal translocations

Graphical abstract

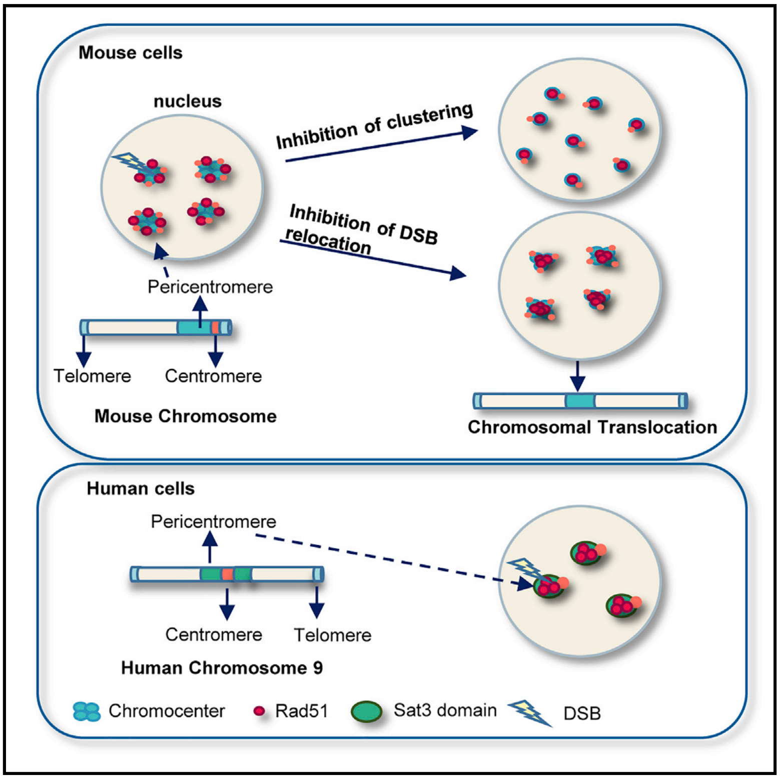

Highlights

- DSBs at human pericentric HC are positionally stable and recruit HR factors
- Repeat clustering restricts Rad51 at the periphery of heterochromatin
- Human pericentromeric HC is more accessible to HR factors than mouse HC
- Inhibition of DSB relocation at clustered repeats leads to translocations

Authors

Ioanna Mitrentsi, Jieqiong Lou, Adèle Kerjouan, John Verigos, Bernardo Reina-San-Martin, Elizabeth Hinde, Evi Soutoglou

Correspondence

e.soutoglou@sussex.ac.uk

In brief

Mitrentsi et al. demonstrate that heterochromatic repeat clustering regulates the spatial activation of DNA repair pathways, and it is not conserved between mouse and human. Clustered repeats are refractory to HR factors, imposing a barrier to illegitimate recombination between repeats from different chromosomes that lead to the formation of translocations.

Mitrentsi et al., 2022, Molecular Cell *82*, 2132–2147
June 2, 2022 © 2022 The Authors. Published by Elsevier Inc.
https://doi.org/10.1016/j.molcel.2022.03.033

CellPress

CellPress
OPEN ACCESS

Molecular Cell

Article

Heterochromatic repeat clustering imposes a physical barrier on homologous recombination to prevent chromosomal translocations

Ioanna Mitrentsi,[1,2,3,4] Jieqiong Lou,[5] Adèle Kerjouan,[5] John Verigos,[7] Bernardo Reina-San-Martin,[1,2,3,4] Elizabeth Hinde,[5,6] and Evi Soutoglou[1,2,3,4,7,8,*]
[1]Institut de Génétique et de Biologie Moléculaire et Cellulaire (IGBMC), Illkirch, France
[2]Institut National de la Santé et de la Recherche Médicale (INSERM), U1258, Illkirch, France
[3]Centre National de Recherche Scientifique (CNRS), UMR7104, Illkirch, France
[4]Université de Strasbourg, Illkirch, France
[5]School of Physics, University of Melbourne, Melbourne, VIC, Australia
[6]Department of Biochemistry and Pharmacology, University of Melbourne, Melbourne, VIC, Australia
[7]Genome Damage and Stability Centre, Sussex University, School of Life Sciences, University of Sussex, Brighton, BN1 9RH, UK
[8]Lead contact
*Correspondence: e.soutoglou@sussex.ac.uk
https://doi.org/10.1016/j.molcel.2022.03.033

SUMMARY

Mouse pericentromeric DNA is composed of tandem major satellite repeats, which are heterochromatinized and cluster together to form chromocenters. These clusters are refractory to DNA repair through homologous recombination (HR). The mechanisms by which pericentromeric heterochromatin imposes a barrier on HR and the implications of repeat clustering are unknown. Here, we compare the spatial recruitment of HR factors upon double-stranded DNA breaks (DSBs) induced in human and mouse pericentromeric heterochromatin, which differ in their capacity to form clusters. We show that while DSBs increase the accessibility of human pericentromeric heterochromatin by disrupting HP1α dimerization, mouse pericentromeric heterochromatin repeat clustering imposes a physical barrier that requires many layers of de-compaction to be accessed. Our results support a model in which the 3D organization of heterochromatin dictates the spatial activation of DNA repair pathways and is key to preventing the activation of HR within clustered repeats and the onset of chromosomal translocations.

INTRODUCTION

Chromatin compaction plays a key role in the choice of DNA repair pathways (reviewed in Clouaire and Legube, 2019; Dantuma and van Attikum, 2016; Mitrentsi et al., 2020). Repetitive DNA elements located at centromeric or pericentromeric chromosomal regions are packaged into heterochromatin, which is highly condensed to prevent unscheduled DNA transactions (Guenatri et al., 2004). Double-stranded DNA breaks (DSBs) in pericentromeric heterochromatin in both mouse and *Drosophila* cells relocate outside of the core heterochromatin domain, presumably to avoid encountering homologous sequences and illegitimate joining (Chiolo et al., 2011; Jakob et al., 2011). In *Drosophila* cells, heterochromatic DSBs relocate to the nuclear pore (Ryu et al., 2015) in an actin- and myosin-dependent manner (Caridi et al., 2018; Schrank et al., 2018). In mouse pericentromeric heterochromatin, DSBs relocate to the periphery of the heterochromatin domain, where they are retained by homologous recombination (HR) factors such as RAD51, whose access into the core of chromocenters is restricted (Tsouroula et al., 2016). The mechanism responsible for restricting the access of HR factors into the core of the pericentromeric heterochromatin domain is unknown. In human cells, pericentromeric heterochromatin has a different spatial organization imposed by distinct biophysical properties. Unlike mouse cells, where identical major satellite repeats from many different chromosomes cluster together to form DAPI-dense chromocenters, human satellite II and satellite III pericentromeric repeats (SatII and SatIII) do not exert the same degree of clustering (reviewed in Brändle et al., 2022) and they do not form chromocenters. It is unclear whether DSB relocation is conserved in human pericentromeric heterochromatin or if alternative mechanisms have evolved to prevent the detrimental recombination between repetitive elements located on different chromosomes.

 © 2022 The Authors. Published by Elsevier Inc.
This is an open access article under the CC BY license (http://creativecommons.org/licenses/by/4.0/).

CellPress
OPEN ACCESS

RESULTS

DSBs in human SatIII pericentromeric heterochromatin remain positionally stable throughout the cell cycle and recruit HR factors

To address whether the DSB repair mechanisms in heterochromatin are conserved in human cells, we generated an experimental system to specifically induce DSBs at human pericentromeric SatIII domains using CRISPR-Cas9 (Figure S1A). Contrary to mouse cells, human pericentric heterochromatin does not correspond to DAPI-dense regions. Thus, to visualize SatIII, which is mainly located on chromosome 9q12 (Figure S1A), we expressed a heat shock factor 1 (HSF1) mutant lacking the activation domain fused to EGFP (DBD-TRIM-EGFP), which constitutively binds to SatIII DNA without activating transcription (Jolly et al., 2002). To induce DSBs at SatIII regions, we expressed Cas9 with SatIII-specific gRNAs (Figures S1A and S1B). As expected, DBD-TRIM-EGFP colocalized with γ-H2AX and 53BP1 (Figures 1A and S1B), which are markers of DNA damage, and it was dependent on the catalytic activity of Cas9 (Figures S1C and S1D). The specific induction of DSBs at SatIII DNA was further confirmed by immuno-FISH (Figure S1E). The number of SatIII domains in U2OS cells was variable between individual cells, with the majority having three to four foci, corresponding to the number of chromosomes 9 contained within each cell (Figure S1F). Cas9-induced DSBs in SatIII resulted in the activation of the DNA damage response (DDR), as exemplified by the phosphorylation of ATM ($pATM^{S1981}$) and the recruitment of MDC1 (Figures S1G and S1H). Notably, the approach did not lead to a massive activation of the DDR, such as that observed when cells were treated with the DNA-damaging agent neocarzinostatin (NCS), further demonstrating that the number of DSBs induced is limited and that the response is localized and not global (Figure S1I).

To determine whether the localization of DSBs generated at SatIII is regulated by the cell cycle, as is the case in mouse pericentric heterochromatin (Tsouroula et al., 2016), we synchronized U2OS cells in either G1/S or G2 phases of the cell cycle and assessed the spatial distribution of γ-H2AX in relation to the SatIII domains using 3D structured illumination microscopy (3D-SIM). Remarkably, and in contrast to mouse cells where γ-H2AX is excluded from heterochromatin in S/G2, we found that γ-H2AX was induced throughout the SatIII core domain independently of the cell cycle stage (Figures 1A–1C and S2A–S2C). These results suggest that in human heterochromatin the Cas9-induced DSBs remain positionally in all stages of the cell cycle and reveal a fundamental difference between human and mouse heterochromatic DSBs.

To investigate whether the distinct DSB localization in human versus mouse heterochromatin is due to differences in the DSB repair mechanisms activated, we assessed in high-resolution the distribution of RAD51 in response to Cas9-induced DSBs at SatIII . As expected, RAD51 was mainly recruited in S/G2 cells (Figures 1D and 1H). Surprisingly, however, and in contrast to what we observed in mouse heterochromatin where RAD51 was at the periphery of the heterochromatic domain promoting DSB relocation (Tsouroula et al., 2016), we found that RAD51 is recruited at the core of the SatIII domains, similar to the γ-H2AX internal pattern (Figure 1D). This was also observed in RPE1 and HeLa cells, excluding the possibility of an artifact due to a specific cell line (Figure S2C).

RPA, which was used as a marker for DNA end resection, was also recruited at the core of the SatIII domains (Figures 1E and 1H), suggesting that DSBs are positionally stable and that the SatIII domains are not refractory to HR. Notably, non-homologous end-joining (NHEJ)-promoting factors, such as 53BP1 and RIF1, were predominantly positioned at the periphery of the SatIII domains (Figures 1G, 1I, S2D, and S2E), suggesting that in the majority of cells in S/G2 there is a spatial separation of repair pathway factors, where HR factors are recruited at the core domain and NHEJ proteins are restricted to the periphery. Consistent with the competition between 53BP1/RIF1 and BRCA1 (Isono et al., 2017), we found a mutually exclusive recruitment pattern between BRCA1 and RIF1 (Figures 1F–1I and S2F). Moreover, the HR factor PALB2 mirroring the BRCA1 pattern was recruited both inside and outside the SatIII domains (Figure S2G), in contrast to mouse heterochromatin where it was recruited only at the periphery (Figure S2H). Cas9-specific DSBs at SatII (mainly located on chromosome 1) (Vourc'h and Biamonti, 2011) led to a similar pattern for γ-H2AX spreading and RAD51 recruitment, demonstrating that our findings can be generalized to other pericentric heterochromatin regions (Figure S2I).

Repeat clustering restricts the access of RAD51 to mouse heterochromatin

As mouse and human pericentric heterochromatin differ in the extent of clustering between repetitive elements, we hypothesized that the use of HR within clustered major satellite repeats in mouse cells could result in aberrant recombination between different chromosomes, leading to translocations. If this is the case, aberrant recombination between repeats of different chromosomes would not be a risk in human cells since repeats do not cluster. To directly assess the role of clustering in DSB relocation and RAD51 exclusion from mouse heterochromatin, we induced DSBs at major satellite repeats in mouse embryonic stem (ES) cells depleted of chromatin assembly factor 1 (CAF1, subunit p150), where chromocenter clustering is disrupted throughout the cell cycle (Houlard et al., 2006) (Figures S3A–S3C). Strikingly, we found that at DSBs induced at the non-clustered major satellite repeats (siCaf1 p150), RAD51 is no longer excluded from the domains (Figures 2A and 2B). This is unlikely due to the loss of heterochromatin marks, as although the size of the chromocenter is markedly reduced and their number is increased (Figure 2C), the repeats still retain H3K9me3 and heterochromatin protein 1 α (HP1α) to a certain extent (Figures 3D and 3E).

Cas9-specific DSBs in supersized chromocenters in mouse cells, generated by the overexpression of the *Drosophila melanogaster* multi-AT-hook satellite DNA-binding protein D1, which was shown to boost clustering (Jagannathan et al., 2018) (Figure 2D), also leads to a very discernible exclusion of RAD51 protein from the core of the heterochromatin domain (Figure 2E).

To investigate whether this phenomenon is observed in other heterochromatic repetitive sequences, we assessed RAD51 localization upon telomeric clustering occurring in ALT (alternative lengthening of telomeres) cells. To experimentally enhance

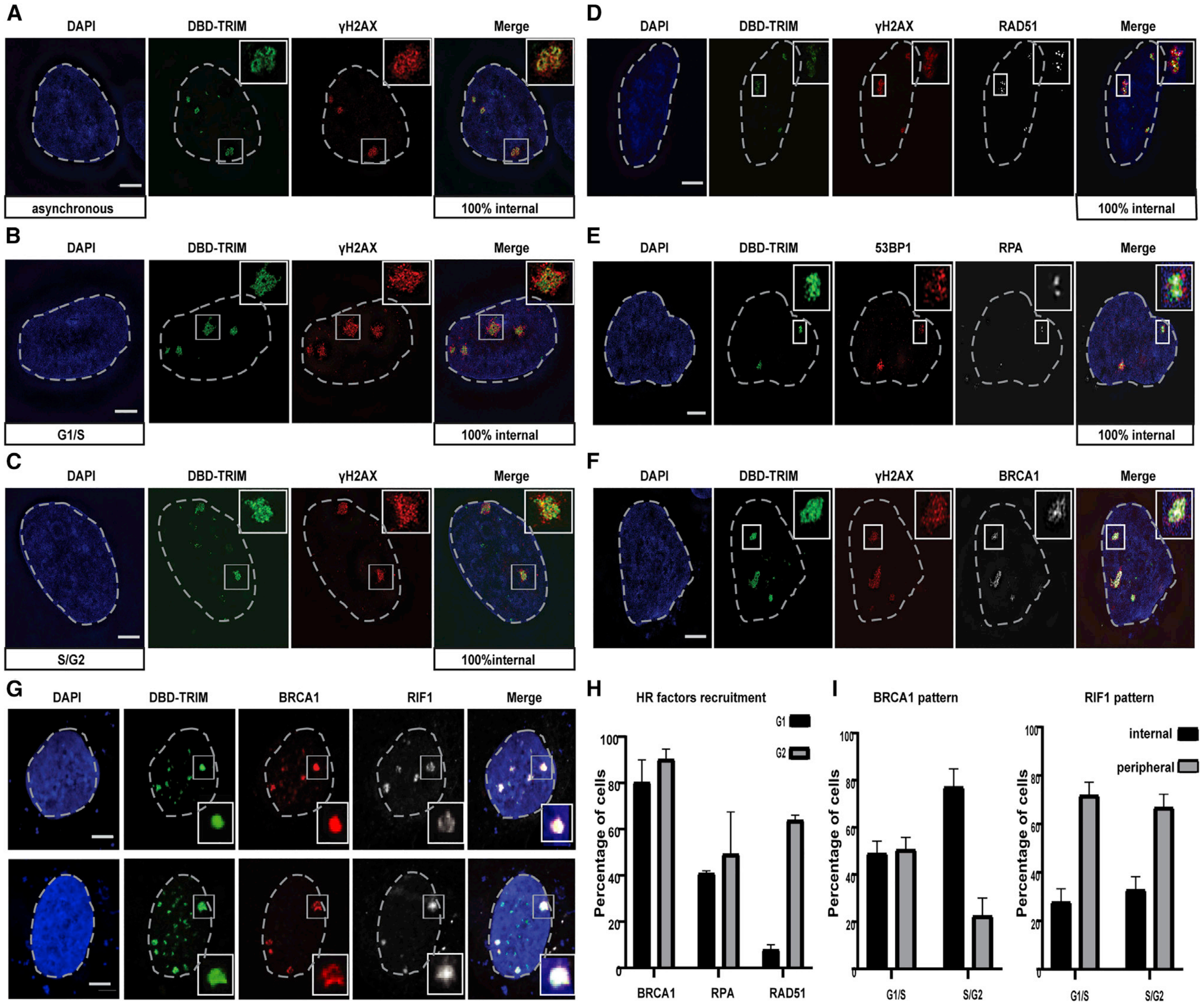

Figure 1. DSBs in SatIII repeats remain positionally stable throughout the cell cycle and recruit HR factors

(A–F) Super resolution (3D-SIM) imaging of U2OS cells expressing DBD-TRIM-EGFP (marker for SatIII domains)+Cas9+gRNA targeting SatIII repeats (SatIII) in (A) asynchronous, (B) G1/S-, and (C) G2-synchronized cells, stained with DAPI and antibodies specific for γ-H2AX (A–C), RAD51 (D), 53BP1 and RPA (E), and BRCA1 (F). 100% internal denotes the percentage of SatIII foci that have the specified DNA repair protein localized within them after DSB induction. RAD51 staining makes small dotty foci.

(G) IF confocal analysis of U2OS cells expressing DBD-TRIM-EGFP+Cas9+gRNA targeting SatIII, stained with DAPI and antibodies specific for BRCA1 and RIF1. Cells in (D)–(G) are asynchronous.

(H) Quantification of RAD51, BRCA1, and RPA recruitment on SatIII domains after DSB induction in U2OS, throughout the cell cycle. For G1, the quantifications are done in EdU and pH3S10 negative cells.

(I) Quantification of BRCA1 and RIF1 patterns on SatIII domains after DSB induction in G1/S or G2-synchronized U2OS cells. Wherever a quantification is not provided, 100% of cells exert the phenotype. Data are represented as mean ± SEM of five experiments of n = 50 cells.

Scale bars represent 5 μm. See also Figures S1 and S2.

telomere clustering, we induced DSBs at telomeres using the FOK1 nuclease fused to the telomeric factor TRF1 (Tang et al., 2013). FOK1 expression induces specific DSBs to all telomeres but leads to their massive clustering only in ALT cells, such as U2OS, and not in HeLa, which are telomerase positive cells (Cho et al., 2014) (Figures 2F and 2H). As expected, DSB induction at telomeres leads to the recruitment of RAD51 in a small fraction of HeLa cells (8%) (Figures 2F, 2G, and 2J) but in 55% of U2OS cells. It is clear, however, that RAD51 colocalizes with the telomeric repeats in HeLa cells, but it is located at the periphery of the clustered telomeric repeats in ALT cells (Figures 2H–2J). Altogether, these results suggest that heterochromatin organization within the nucleus has fundamental implications in the spatial distribution of DNA repair pathways. This notion argues against the need for directed motion along nuclear filaments for DSBs to relocate away from heterochromatin (HC).

CellPress
OPEN ACCESS

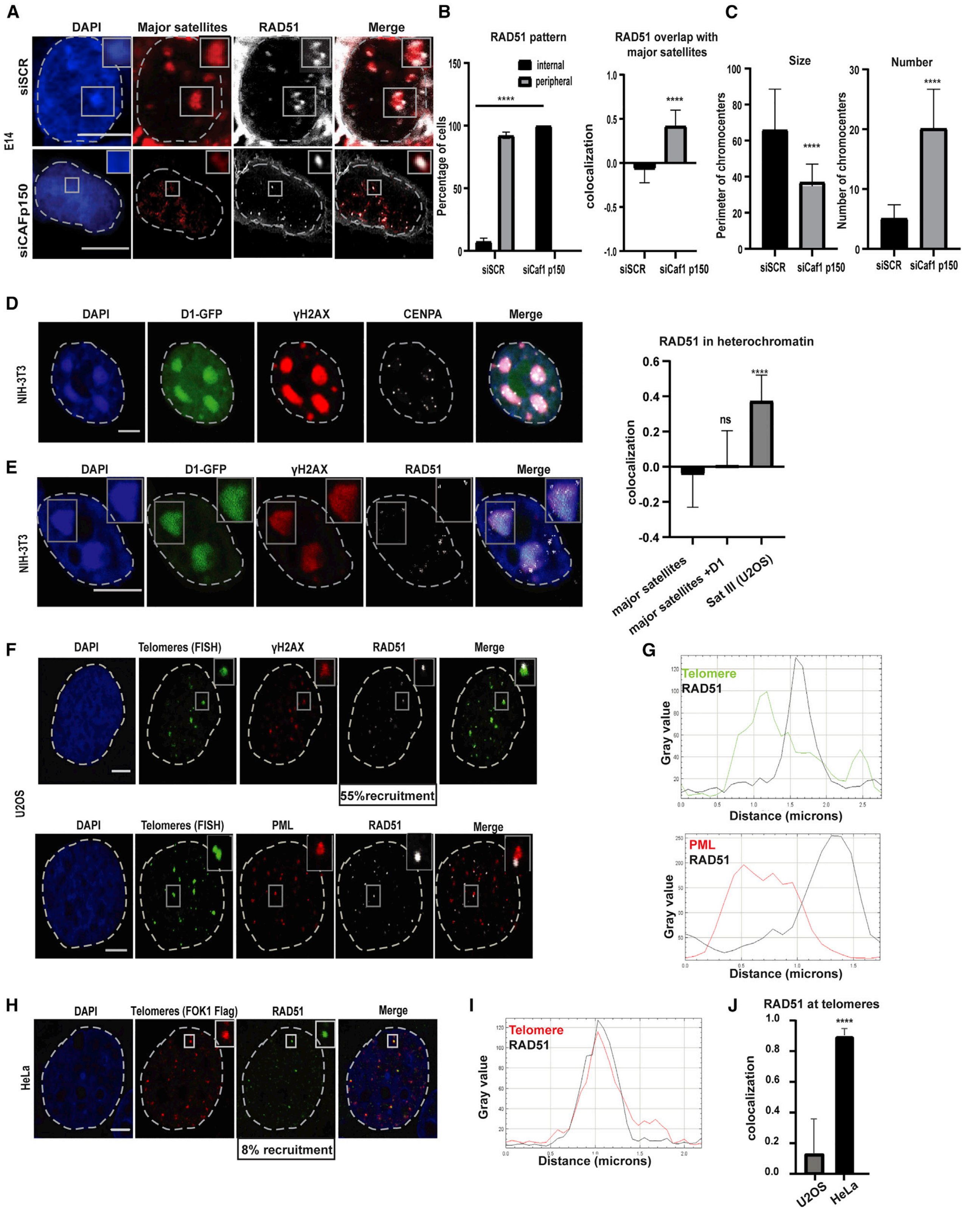

(legend on next page)

Indeed, we found that DSB relocation was independent of an actin- or myosin-related process, as depletion of the actin-related proteins ARP2 (Figures S3F, S3G, and S3I), ARP3 (Figures S3F–S3H), and UNC45 (Figures S3F, S3G, and S3J) did not alter DSB distribution in mouse pericentric heterochromatin (Figures S3F–S3J), contrary to what has been previously suggested (Caridi et al., 2018).

DSBs increase the accessibility of human SatIII heterochromatin to HR factors

In cells, RAD51 does not diffuse alone but travels with BRCA2/PALB2/BRCA1 in a nearly 2MDa multimeric complex (Marmorstein et al., 2001) in which BRCA2 oligomerizes and each BRCA2 monomer binds to several RAD51 molecules (Jensen et al., 2010). This multimeric assembly, which spans up to several hundred nanometers, can scan the nuclear volume and effectively deliver RAD51 to single stranded (ss) DNA generated by DNA damage. Heterochromatin is among the densest nuclear compartments and impedes the diffusion of macromolecules in a size-dependent manner (Bancaud et al., 2009). To investigate whether repeat clustering impedes the access of the BRCA2/PALPB2/BRCA1/RAD51 complex to the heterochromatic domains, we transiently expressed mCherry-BRCA2 or PALB2-GFP in both mouse and human cells and measured their accumulation/exclusion from mouse chromocenters or human SatIII domains, in the absence of DSBs (Figures 3A and 3B). We found that although the intensity of BRCA2 and PALB2 inside the SatIII domains (marked by DBD-TRIM) is similar or enhanced compared with the nucleoplasm, BRCA2 and PALBP2 are markedly depleted from mouse heterochromatin (marked with HP1α and/or DAPI), (Figures 3A and 3B), suggesting that the complex can enter more easily in human heterochromatin than mouse heterochromatin (Figures 3A and 3B). In contrast, XRCC4, an NHEJ factor, exerted a better ability to access mouse heterochromatin, which was comparable with the inert protein mCherry (Figures 3C and 3D).

To monitor the accessibility of mouse versus human heterochromatin toward molecular diffusion in real time, and observe whether it changes after DNA damage, we employed pair correlation function (pCF) analysis (Digman et al., 2008), an approach of fluorescence fluctuation spectroscopy (FFS), which has the capacity to measure the time it takes an inert protein (mKate2–26 kDa) to diffuse into fluorescently labeled nuclear structures (Hinde et al., 2010). Using pCF, we spatially cross-correlated the fluctuations in mKate2 fluorescence intensity outside versus inside of either mouse (HP1) (Figures 4A–4C) or human (DBD-TRIM) (Figures 4D–4F) heterochromatin in G1 and G2 and in the presence or absence of DNA damage (Figures 4A–4F). In mouse cells, the heterochromatin domain in G1 and G2 was found to present as an obstacle to free mKate2 diffusion that imparted a delayed characteristic entry time, which extended from ~5 to10 ms and did not significantly change upon DSB induction (Figures 4A–4C). In human cells, SatIII domains in G1 were more accessible toward mKate2 diffusion on the timescale of the experiment than in G2; interestingly, in the presence of Cas9-induced DSBs in G2, where HR is predominant, mKate2 diffusion into SatIII domains becomes more rapid, with a significant shift in the characteristic entry time from ~20 to ~5–10 ms (Figures 4D–4F). Collectively, this result reveals that Cas9-induced DSBs increase the accessibility of the human SatIII domains, specifically in G2.

To next assess whether the measured accessibility of heterochromatic DSBs is reflected in the potential of HR and NHEJ factors (PALB2 and XRCC4, respectively) to enter and diffuse within these fluorescently labeled nuclear structures, we employed pCF alongside an autocorrelation function (ACF) analysis (Figures 4G–4R). We found that the characteristic arrival time of PALB2 into heterochromatic DSBs was slower compared with XRCC4, which is consistent with the fact that PALB2 diffuses as part of a larger nuclear complex (Figures 4I, 4K, 4O, 4Q, S4A–S4D). More specifically, in mouse heterochromatin, PALB2 diffusion is markedly delayed throughout the cell cycle, and the presence of DSBs did not change its characteristic arrival time (Figures S4A and S4B). In human cells, however, DSBs in SatIII domains in G2 lead to a shift in the arrival time that is weighted toward a shorter time scale, as observed for mKate2 (Figures S4C and S4D). Collectively, we interpret this shift to reflect an increase in access overlaid with the binding dynamics of PALB2 at DSBs.

To confirm this interpretation, we next, using ACF, autocorrelated the fluctuations in PALB2-GFP and mCherry-XRCC4 fluorescence intensity outside versus inside both mouse and human

Figure 2. Repeat clustering excludes RAD51 from heterochromatic DSBs

(A) Immuno-FISH confocal analysis of WT E14 cells (siScr) or E14 cells depleted of Caf1 p150 (siCAF1p150) expressing Cas9+gRNA targeting major satellite repeats, stained with DAPI, a major satellite probe and RAD51-specific antibody.

(B) Quantification of RAD51 pattern and cell profiler analysis of RAD51 colocalization with major satellite repeats on either WT E14 cells or E14 cells depleted of Caf1 p150.

(C) Cell profiler analysis of chromocenters' size (perimeter) (left) and quantification of chromocenters' number (right) in WT E14 cells or E14 cells depleted of Caf1 p150, based on the major satellite FISH signal.

(D and E) IF confocal analysis of NIH-3T3 cells expressing (D) D1-GFP, stained with DAPI and CENPA, and (E) D1-GFP+Cas9+gRNA targeting major satellite repeats, stained with DAPI and antibodies specific for γH2AX and RAD51. Cell profiler analysis of RAD51 colocalization with chromocenters on NIH-3T3 cells ± D1-GFP expression, compared with SatIII domains in U2OS cells.

(F and G) Immuno-FISH confocal analysis of U2OS cells expressing the Flag-TRF1-FOK1 endonuclease, stained with DAPI, telomeric probe (PNA), and antibodies specific for γH2AX, PML, and RAD51 (F), and line scan analysis or RAD51 colocalization with telomeres or PML bodies (G).

(H and I) IF confocal analysis of HeLa cells expressing Flag-TRF1-FOK1-Flag, stained with DAPI, Flag, and RAD51-specific antibodies (H), and line scan analysis of RAD51 colocalization with telomeres (I).

(J) Cell profiler analysis of Rad51 colocalization with telomeres on U2OS versus HeLa cells. Wherever a quantification is not provided, 100% of cells exert the phenotype.

Scale bars represent 5 μm. Data are represented as mean ± SEM of three experiments with n = 50 cells. See also Figure S3.

CellPress
OPEN ACCESS

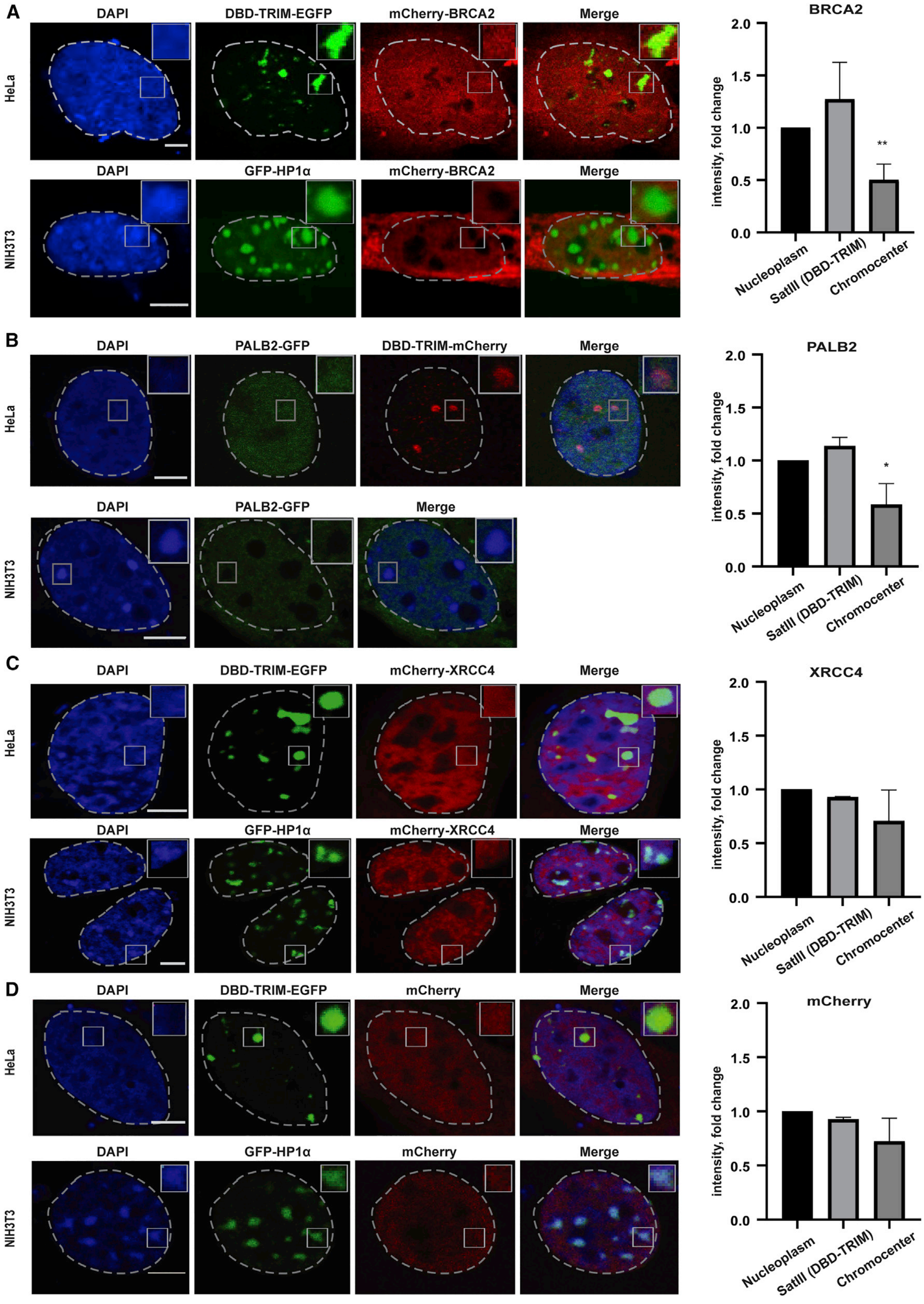

(legend on next page)

heterochromatin DSBs and calculated the ratio of moving molecules that distribute between these two environments in the presence or absence of DNA damage (Figures 4J, 4L, 4P, 4R, and S4E–S4H). Interestingly, the ratio of moving molecules in mouse heterochromatin is up to 6-fold higher for XRCC4 than PALB2 in the presence and absence of DNA damage, demonstrating once more the difficulty for HR factors to enter and interact with mouse heterochromatin (Figures 4J and 4L). Notably, only DSBs in human cells in G2 enable a significant increase in entry of PALB2 factor in SatIII domains (Figure 4R). These results, altogether, suggest that Cas9-induced DSBs could increase the accessibility of the human SatIII domain to HR factors specifically in G2, in contrast to the mouse chromocenters, and that the size of the BRCA2/PALB2/BRCA1/RAD51 complex may render its accessibility more sensitive to the diffusion barriers imposed by clustering.

DSBs trigger an increase in Hp1α dimerization in mouse heterochromatin but decrease HP1 dimer formation in human SatIII domains

The formation of silenced and condensed heterochromatin involves the enrichment of HP1. We have previously demonstrated that DSBs in mouse pericentric heterochromatin lead to an increase in the level of H3K9me3 and all HP1 isoforms, consistent with the persistent barrier observed above (Tsouroula et al., 2016). Interestingly, however, DSBs at SatIII in human cells did not show an increase in HP1α or HP1β and resulted in the partial eviction of HP1γ (Figures S5A and S5B). These results further support the notion that there are fundamental differences between human and mouse cells and that DSBs in human pericentric heterochromatin lead to increased accessibility of the domain. The ability of an inert protein to enter heterochromatin (Strom et al., 2017) and thus the permeability of heterochromatin have been correlated with the density of the HP1α protein at the domain boundary (Hinde et al., 2015; Strom et al., 2017). Indeed, it has been shown that HP1α is not equally partitioned in these domains but that it exists as monomers at the core and as a dimer at the boundary, generating a physical barrier (Hinde et al., 2015; Larson et al., 2017; Strom et al., 2017). To determine whether the differences in diffusion we observed correlate with the presence or absence of HP1α dimers, we employed another FFS-based method called number and brightness (NB), which has the capacity to spatially map the oligomeric state of fluorescently tagged HP1α (Digman et al., 2008). To this end, we quantified the fraction of HP1α dimers in mouse chromocenters and human SatIII domains in G1 and G2 in the presence or absence of Cas9-induced DSBs. Based on an HP1α monomer calibration we found that HP1α dimerizes at the boundary of both chromocenters and SatIII domains (Figures 5A and 5B). DSBs at SatIII in G1 or mouse heterochromatin in both G1 and G2 resulted in an increase of the density of HP1α dimers (Figures 5C and 5D). Interestingly, and consistent with the increased diffusion of mKate2 and PALB2 in human SatIII versus the mouse chromocenters, DSBs at human SatIII domains in G2 resulted in a significant decrease in the density of HP1α dimers at the boundary of the domain (Figure 5D). These results reveal an anti-correlation between HP1α dimerization and protein diffusion or HR factor recruitment at the core of SatIII domains. To investigate whether HP1α dimers render mouse heterochromatin inaccessible to HR factors, we depleted the three isoforms of HP1 (α, β, and γ) (Tsouroula et al., 2016) and complemented cells with HP1α or the Hp1α^{I165E} mutant, which cannot dimerize, and tested the potential of RAD51 and BRCA1 to diffuse inside the chromocenters. As we have shown previously (Tsouroula et al., 2016), simultaneous depletion of all HP1 isoforms decreased the percentage of cells that recruited RAD51 and BRCA1 at pericentric DSBs (Figures 5E–5G and S5C), consistent with the role of HP1 in DNA end resection (Soria and Almouzni, 2013). Interestingly, this effect was dependent on HP1 dimerization (Figures 5F, 5G, and S5D), suggesting that HP1 dimerization is important for DNA repair. Remarkably, however, in those cells in which RAD51 and BRCA1 were recruited, they were restricted to the periphery of the chromocenters in G2 (Figures 5F, 5G, and S5E). Similar results were obtained in SUV3-9 1 and 2 double knockout (KO) (MEFs) in which HP1α is not bound at chromocenters because of the lack of H3K9me3 (Figures S5F–S5I). These results are consistent with the observation that in SUV3-9 KO MEFs, chromocenter accessibility to an inert protein is similar to wild-type MEFs (Erdel et al., 2020) and suggest that in mouse heterochromatin, additional mechanisms are at play to establish a diffusion barrier. Conversely, in human cells, depletion of HP1α reduced the characteristic arrival time of mKate2, suggesting that HP1 is sufficient to create a diffusion barrier in human heterochromatin (Figure S5J). Concomitantly, depletion of all isoforms of HP1 in human cells resulted in an increase of the accessibility of the SatIII domains to BRCA1 in G1 (Figure S5I).

Artificial increase of accessibility in mouse heterochromatin permits access to HR proteins

It has been shown that the tethering of a strong transcriptional activator (VPR) to mouse heterochromatin disrupts interactions between its segments and renders the domain accessible to diffusion (Erdel et al., 2020). To investigate the behavior of HR proteins under de-condensed heterochromatin conditions, we induced DSBs at chromocenters while simultaneously tethering VPR with catalytically inactive Cas9 (EGFP-dCas9-VPR) in mouse NIH-3T3 cells. This resulted in chromocenter decompaction and disappearance of DAPI-dense regions (Figure 6A). Surprisingly, under these conditions, RAD51 and BRCA1 were visualized for the first time at the core of the domain

Figure 3. Accumulation of HR and NHEJ factors into mouse and human heterochromatin foci

(A) IF confocal analysis of NIH-3T3 cells expressing EGFP-HP1α+mCherry-BRCA2 or HeLa cells expressing DBD-TRIM-EGFP+mCherryBRCA2.

(B) IF confocal analysis of NIH-3T3 cells expressing PALB2-GFP or HeLa cells expressing DBD-TRIM-mCherry+PALB2-GFP.

(C and D) IF confocal analysis of NIH-3T3 cells expressing EGFP-HP1α and mCherryXRCC4 (C), or mCherry (D) and HeLa cells expressing mCherry-XRCC4 (C) or mCherry (D). Intensity quantifications of mCherry-BRCA2, PALB2-GFP, mCherry-XRCC4, and mCherry in heterochromatin area versus a similar size area in the nucleoplasm is represented by a fold change.

Scale bars represent 5 μm. Data are represented as mean ± SEM.

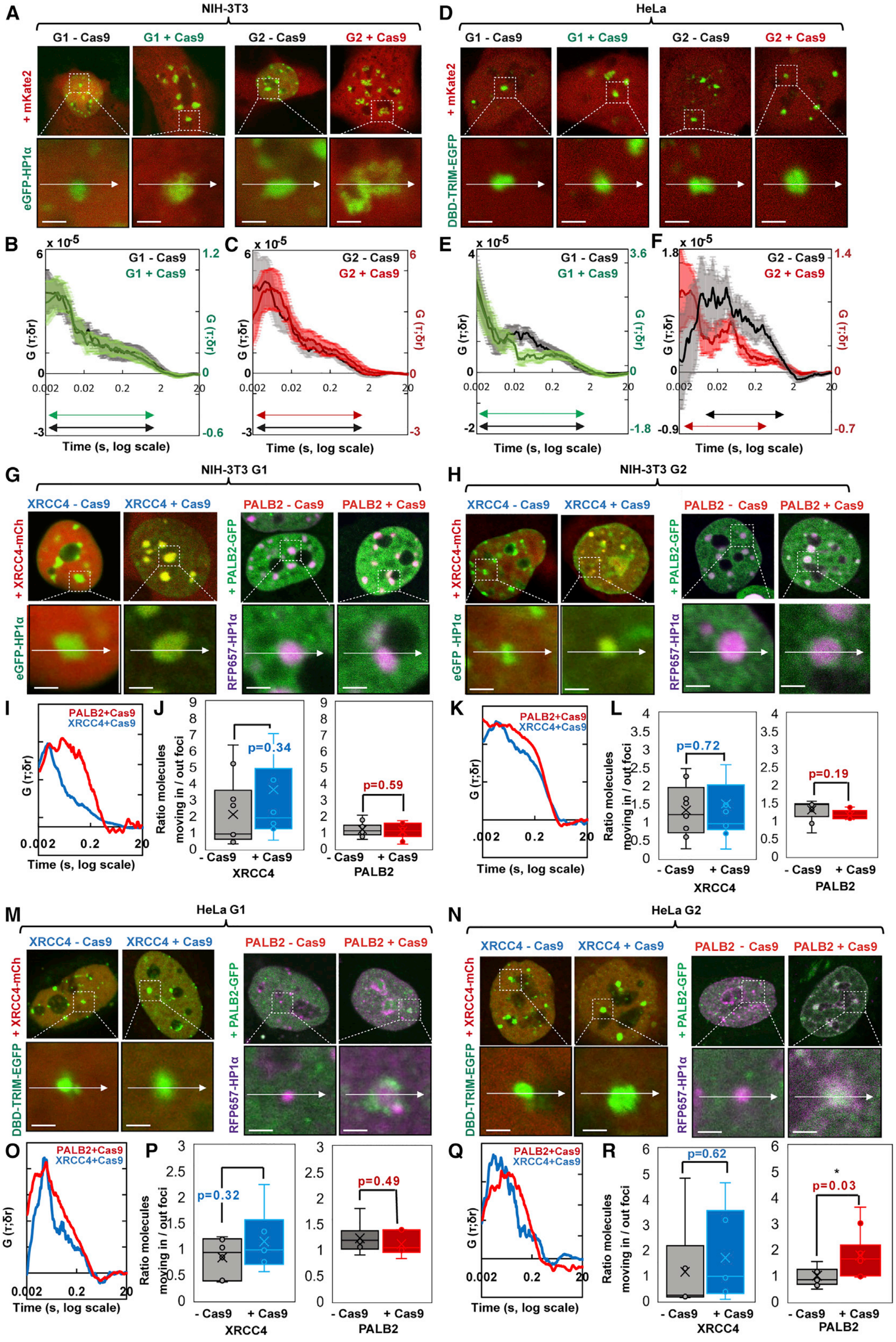

(legend on next page)

(Figures 6B and 6C). The same pattern was observed in super-clustered chromocenters upon tethering of VPR (Figure 6D). In accordance with these results, when we measured the capacity of BRCA2 to accumulate in the chromocenters under the same conditions, we observed an increased intensity compared with the nucleoplasm, similar to the inert protein mCherry (Figures 6E–6G), suggesting that mouse chromocenter clustering makes the domain inaccessible and that it requires extreme de-condensation for HR factors to enter. Contrary to this, human SatIII domains, which do not cluster, are more accessible, and in this case, HP1α dimers are sufficient to establish a barrier and control the accumulation/exclusion of proteins. It appears then that in mouse cells, several layers of protection are at play to limit protein diffusion inside chromocenters.

Repeat clustering prevents recombination between repeats and the onset of chromosomal translocations

To investigate the functional significance of chromocenter clustering in imposing a barrier to prevent deleterious recombination events between different chromosomes, we forced the entry of RAD51 to the core of the chromocenters by tethering the BRC3 domain of BRCA2 via dCas9 (EGFP-dCas9-BRC3), which recruits RAD51 (Tsouroula et al., 2016). This resulted in the recruitment of RAD51 inside the core domain (Tsouroula et al., 2016) and in the subsequent stabilization of the breaks inside the chromocenters (Figure 7A). To determine the functional consequence of the lack of DSB relocation and RAD51 internalization, we scored for chromosomal translocations originating at chromocenters on metaphase spreads by FISH, using probes specific for major satellite repeats and telomeres. Chromosomal translocations originating from the pericentric repeats of two different chromosomes would be expected to lead to the formation of metacentric chromosomes with a single pericentromere and lacking the short arm telomeres. We found that inhibition of DSB relocation led to a 5-fold increase in the frequency of translocations (Figures 7A–7C). Interestingly, induction of super clustering by D1 overexpression led to a 10-fold increase in the percentage of translocations forming between pericentric repeats, further demonstrating the toxic role of repeat proximity in promoting illegitimate recombination between repeats of different chromosomes leading to translocations (Figures 7A–7C). In line with this observation, disruption of clustering by Caf1 depletion in mouse ES cells reduced the number of RAD51-dependent chromosomal translocations when BRC3 was tethered at the major satellite repeats (Figures 7D and 7E). All these events are dependent on RAD51 activity, as translocations were reduced in the presence of a catalytic inhibitor of RAD51 (Figure 7C). Overall, these results suggest that DSB relocation and RAD51 exclusion from mouse chromocenters are of fundamental importance to avoid illegitimate recombination between clustered repetitive elements and to prevent the onset of potentially oncogenic chromosomal translocations.

DISCUSSION

Repetitive DNA is often packaged in heterochromatin to maintain its stability. Maintaining the integrity of pericentromeric repetitive sequences is key for proper chromosome segregation and cell fitness. Despite the substantial progress in our understanding of how repetitive elements maintain their integrity when they are damaged from work in model organisms (Caridi et al., 2018; Chiolo et al., 2011; Hansen et al., 2016; Lambert et al., 2010; Schrank et al., 2018; Tsouroula et al., 2016), whether these mechanisms are entirely conserved in human cells was unknown. Here, we have developed a CRISPR-Cas9 system to specifically induce and visualize DSBs in human pericentric heterochromatin. We demonstrate that the spatial regulation of DNA repair pathway choice in heterochromatin is not conserved in human cells and that in contrast to previous observations that HR does not take place at the core of pericentric heterochromatin and that DSBs relocate away from the domain (Tsouroula et al., 2016) in mouse and *Drosophila*, DSBs in human SatIII

Figure 4. Spatial evolution of mKate2 diffusion into mouse versus human heterochromatin foci and quantification of the impact this has on PALB2 versus XRCC4 entry

(A) Confocal images of NIH-3T3 cells synchronized in G1 or G2, expressing mKate2+EGFP-HP1α in the absence of Cas9 or with Cas9+gRNA targeting major satellite repeats, alongside the region of interest, where a line scan (white arrow) was selected for pCF analysis, enlarged (bottom row).

(B and C) pCF 6–8 (δr = 6–8 pixels) analysis of mKate2 accessibility into NIH-3T3 G1 (B) and G2-arrested cells (C), heterochromatin before (gray) versus after (G1, green; G2, red) DSB induction (n = 6–10 measurements, n ≥ four cells, two biological replicates). The pCF peak represents the characteristic arrival time (most frequent) and in the case of passive diffusion it can be either a tau = 0 if the molecules are fast or tau > 0 if there is a delay in arrival. The width of the peak represents how passive the transport process is, and if there is a long tail of times (shoulder) this is likely due to lack of accessibility.

(D) Confocal images of HeLa cells synchronized in G1 or G2, expressing mKate2+DBD-TRIM-EGFP in the absence of Cas9 or with Cas9+gRNA targeting SatIII, with the region of interest, where a line scan was selected for pCF analysis.

(E and F) pCF 6–8 (δr = 6–8 pixels) analysis of mKate2 access into HeLa G1 (E) and G2 (F) heterochromatin before (gray) versus after (G1, green; G2, red) DSB induction (n = 10–14 measurements, n ≥ 6 cells, two biological replicates).

(G and H) Confocal images of NIH-3T3 cells synchronized in G1 (G) or G2 (H), expressing mCherry-XRCC4+EGFP-HP1α or PALB2-GFP+RFP657-HP1α in the absence of Cas9 or with Cas9+gRNA targeting major satellite repeats, with the region of interest, where a line scan was selected for pCF analysis, enlarged.

(I–L) pCF 6–8 (δr = 6–8 pixels) analysis of XRCC4 (blue) versus PALB2 (red) access into NIH-3T3 G1 (I) or G2 (K) heterochromatin after DSB induction (n = 6–12 measurements, n ≥ 5 cells, two biological replicates) and ACF (δr = 0 pixels) analysis of the ratio of moving XRCC4 (left) versus PALB2 (right) molecules inside versus outside NIH-3T3 G1 (J), or G2 (L) heterochromatin before (gray) and after (blue or red) DSB induction.

(M and N) Confocal images of HeLa cells synchronized in G1 (M) or G2 (N) expressing XRCC4-mCherry+DBD-TRIM-EGFP or PALB2-GFP+ RFP657-HP1-α in the absence of Cas9 or with Cas9+gRNA targeting SatIII, with the region of interest, where a line scan was selected for pCF analysis, enlarged.

(O–R) pCF 6–8 (δr = 6–8 pixels) analysis of XRCC4 (blue) versus PALB2 (red) access into HeLa G1 (O) or G2 (Q) heterochromatin after DSB induction (n = 8–10 measurements, n ≥5 cells, two biological replicates) and ACF (δr = 0 pixels) analysis of the ratio of moving XRCC4 (left) versus PALB2 (right) molecules inside versus outside HeLa G1 (P) or G2 (R) heterochromatin foci before (gray) and after (blue or red) DSB induction.

Scale bars represent 2 μm. Data are represented as mean ± SEM.See also Figure S4.

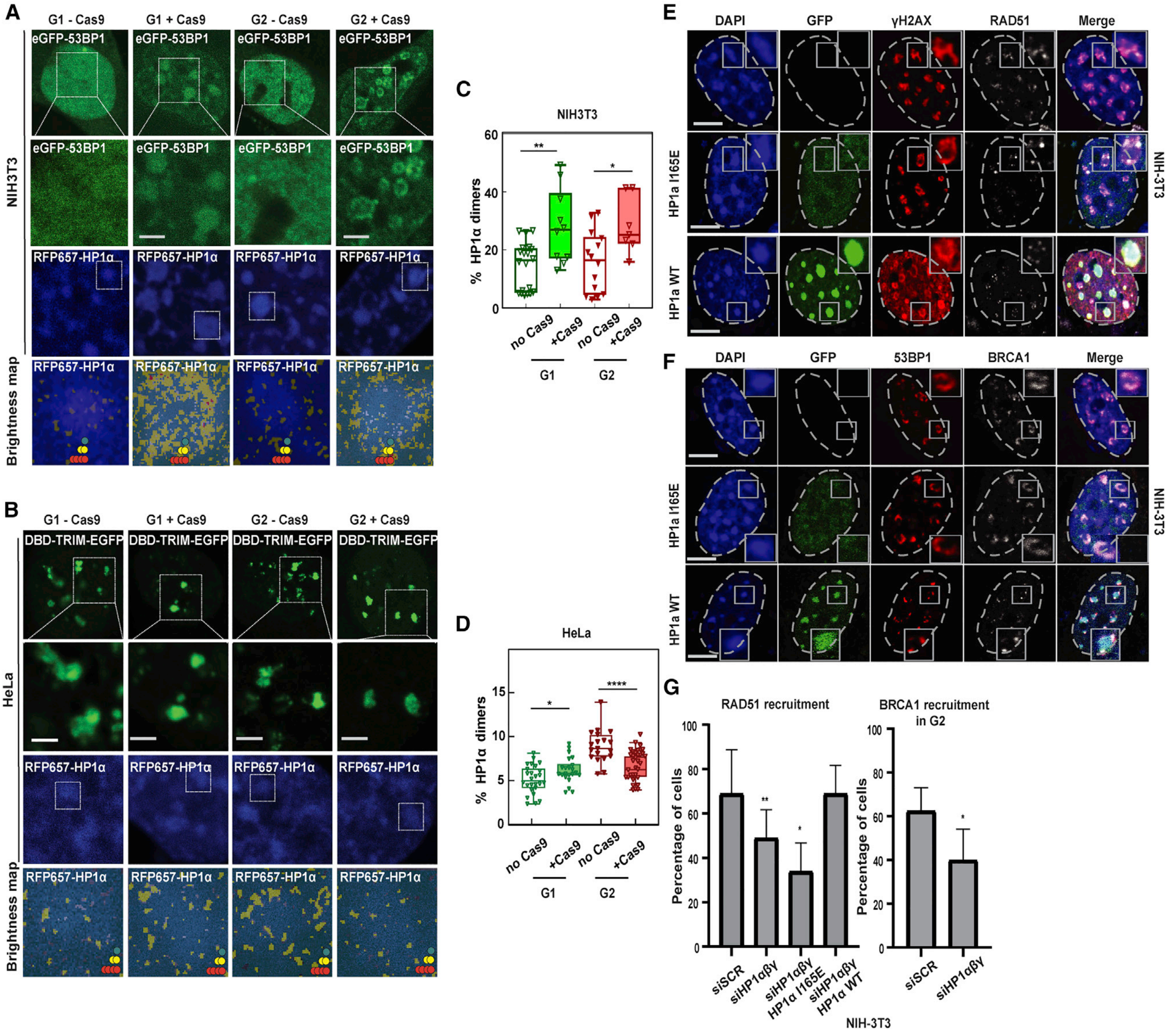

Figure 5. Hp1α dimerization in heterochromatin in the absence or presence of DSBs and its role in RAD51 recruitment

(A and B) Confocal images of (A) NIH-3T3 cells synchronized in G1 or G2, expressing 53BP1-eGFP+RFP657-HP1α in the absence of Cas9 or with Cas9+gRNA targeting major satellite repeats, or (B) HeLa cells synchronized in G1 or G2, expressing DBD-TRIM-EGFP+RFP657-HP1α in the absence of Cas9 or with Cas9+gRNA targeting SatIII, as well as the region of interest from which a number and brightness (NB) frame scan acquisition was recorded (top three rows), alongside an overlay of HP1α intensity with the NB map of HP1α monomer (dark green spots), dimer (yellow spots), and oligomer localization (red spots) (bottom row).

(C and D) NB quantitation of the percentage of RFP657-HP1α dimer present in (C) NIH-3T3 (n = 7–20 measurements, n ≥ 6 cells, three biological replicates) or (D) HeLa (n = 19–35 measurements, n ≥ 12 cells, three biological replicates) heterochromatin in the absence versus presence of Cas9 in G1 versus G2. Box and whisker plots show mean line and all data points.

(E–G) IF confocal analysis of G2 synchronized NIH-3T3 cells expressing Cas9+gRNA targeting major satellite repeats, depleted of Hp1αβγ and complemented with EGFP-HP1α^{I165E} or GFP-HP1α, stained with DAPI and antibodies specific for γ-H2AX and RAD51 (E) or 53BP1 and BRCA1 (F) and (G) quantification of RAD51 (left) and BRCA1 (right) recruitment.

Scale bars represent 5 μm. Data are the mean ± SD of three experiments with n = 50 cells. See also Figure S5.

remain positionally stable throughout the cell cycle and they can be repaired by HR within the heterochromatin domain.

In mice, all chromosomes are acrocentric and contain very long stretches of major satellite repeats at the tip of each chromosome, which cluster and form chromocenters. In human cells, however, pericentromeric repeats are mainly in the middle of metacentric chromosomes, are variable in sequence and length, and even though they might stochastically associate in a population of cells (Alcobia et al., 2003; Guenatri et al., 2004; Swanson et al., 2013; Weierich et al., 2003; Wiblin et al., 2005), they were

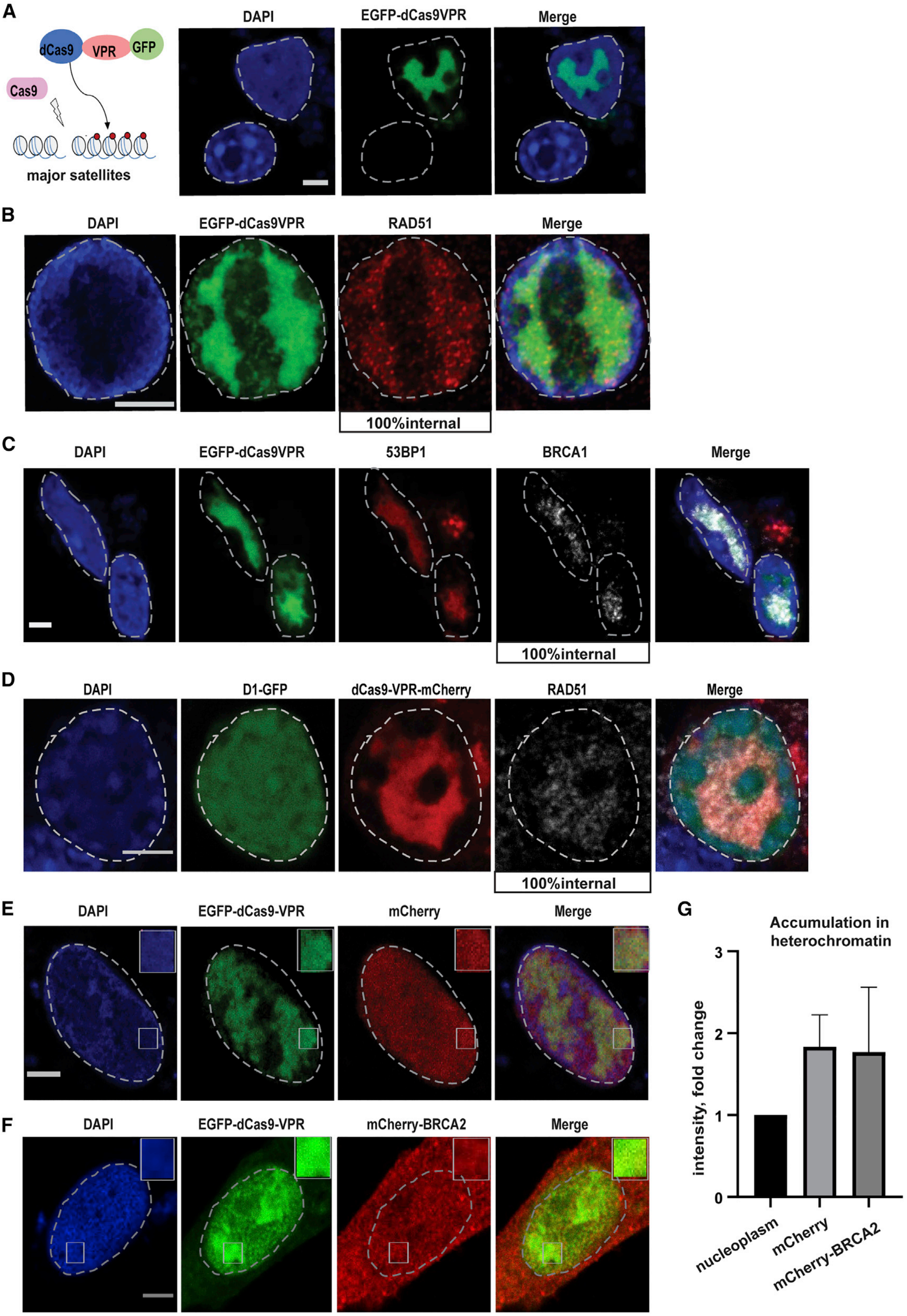

(legend on next page)

CellPress
OPEN ACCESS

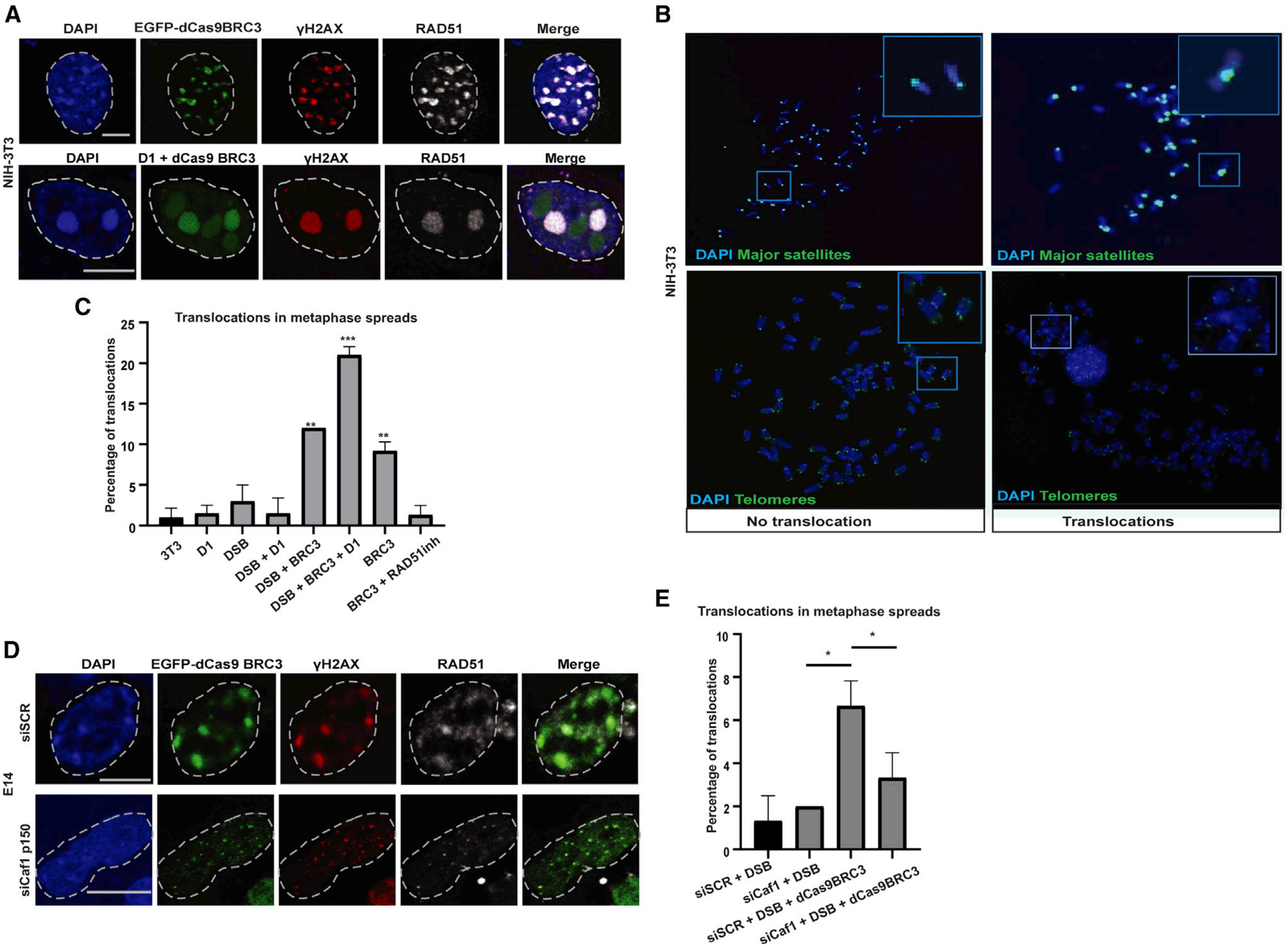

Figure 7. Repeat clustering imposes a physical barrier to prevent homologous recombination between repeats and chromosomal translocations

(A) IF confocal analysis of NIH-3T3 cells expressing EGFP-dCas9-BRC3+gRNA targeting major satellite repeats, or EGFP-dCas9-BRC3+D1-GFP+gRNA targeting major satellite repeats, stained with DAPI and antibodies specific for γ-H2AX and RAD51.

(B) Representative confocal image of metaphase spreads in cells expressing EGFP-dCas9-BRC3, after FISH for major satellite repeats or telomeres (PNA) and stained with DAPI. The insets depict chromosomal translocations.

(C) Quantification of the percentage of translocations in cells expressing D1-GFP or a gRNA targeting major satellite repeats in the following conditions: together with dCas9/ Cas9/ Cas9+D1/ Cas9-BRC3/ Cas9-BRC3+D1/ EGFP-Cas9-BRC3 and treated with DMSO or RAD51 inhibitor (RAD51i).

(D and E) (D) IF confocal analysis of WT E14 cells or depleted of Caf1 p150, expressing EGFP-dCas9-BRC3+gRNA targeting major satellite repeats, stained with DAPI and antibodies specific for γ-H2AX and RAD51, and (E) quantification of the number of translocations. Images are representative of 3–7 experiments. Wherever a quantification is not provided, 100% of cells exert the phenotype.

Scale bars represent 5 μm. Data are the mean ± SD of at least three experiments with n = 50 metaphases.

not shown to cluster to the degree that mouse pericentromeric repeats do. In accordance with this, although a substantial portion of the human genome consists of AT-rich satellite DNA repeats, human cells exhibit almost no DAPI-dense nuclear foci (Brändle et al., 2022). In line with the correlation between repeat clustering and DSB relocation, DSBs at nucleolar repeats

Figure 6. Extreme de-condensation is required to increase accumulation of HR factors in the major satellite repeats area

(A) IF confocal analysis of (A) NIH-3T3 cells expressing EGFP-dCas9-VPR+gRNA targeting major satellite repeats stained with DAPI.

(B and C) NIH-3T3 cells expressing EGFP-dCas9-VPR+Cas9+gRNA targeting major satellite repeats stained with DAPI and antibodies specific for RAD51 or BRCA1.

(D) NIH-3T3 cells expressing mCherry-dCas9-VPR+D1-GFP+Cas9+gRNA targeting major satellite repeats, stained with DAPI and RAD51 specific antibody.

(E–G) IF confocal analysis of NIH-3T3 cells expressing EGFP-dCas9-VPR+gRNA targeting major satellite repeats +mCherry (E) or mCherry-BRCA2 (F) and quantification of mCherry or mCherry-BRCA2 intensity in heterochromatin area versus the nucleoplasm (G). Heterochromatin is visualized by the EGFP-dCas9-VPR or mCherry-dCas9-VPR accumulation in major satellites. Wherever a quantification is not provided, 100% of cells exert the phenotype.

Scale bars represent 5 μm. Data are the mean ± SD of three experiments with n = 50 cells.

that cluster at the nucleolus relocate to nucleolar caps, and RAD51 associates only with the nuclear caps and does not enter the nucleolus (Harding et al., 2015; Larsen et al., 2014; van Sluis and McStay, 2015; Warmerdam et al., 2016). In further agreement, DSBs at telomeres of ALT cells, which cluster at the PML bodies, are refractory to the entrance of RAD51. Moreover, disruption of clustering leads to the recruitment of RAD51 inside the chromocenter and the DSBs do not relocate. Interestingly, centromeres of acrocentric chromosomes have been proposed to cluster around the nucleolus (Weierich et al., 2003). It would, therefore, be interesting to investigate whether the degree of centromeric clustering correlates with RAD51 localization in the presence of centromeric DSBs and whether boosting of clustering leads to chromosomal translocation involving the centromeres.

We hypothesized that repeat clustering builds a refractory environment for HR, which can otherwise lead to translocations by illegitimately joining identical repeats from different chromosomes. In agreement with this hypothesis, we demonstrated that inhibition of the DSB relocation led to an increase in the number of translocations originating from these repeats. Boosting of clustering further increased the number of translocations and disruption of clustering decreased them, demonstrating that bringing more repeats in close proximity increases their propensity to translocate to each other in the presence of DNA damage. These results are in line with observations in *Drosophila* cells where inhibition of DSB relocation results in genomic instability (Ryu et al., 2015).

To investigate the mechanism behind the refractory nature of clustered repeats, we assessed how accessible these domains are, whether they build barriers to protein diffusion, and whether these features change after DNA damage. We found that clustered repeats in mouse chromocenters pose a stronger barrier to protein diffusion than in human chromocenters and that DNA damage further increased the accessibility of human heterochromatin. This diffusion barrier correlated with the presence of HP1α dimers at the periphery of heterochromatin and not with the presence of HP1γ. Different scenarios can be envisaged for the role of Hp1a dimerization in the regulation of the HR at the periphery of the heterochromatin. In one of them, HP1 dimers are formed to block the access of BRAC2/PALB2/BRCA1/RAD51 by increasing local chromatin condensation. This leads to a spatial separation of DNA end resection and strand invasion and therefore forces the resected DNA ends to relocate and become stabilized at the periphery by strand invasion and RAD51 loading. The HP1 dimers can form between preexisting HP1 molecules or between preexisting and *de novo* recruited HP1 molecules to DSBs.

Surprisingly, HP1 depletion decreased the recruitment of RAD51 to heterochromatic DSBs but did not affect its localization at the periphery of mouse heterochromatin. It is, therefore, possible that although the increase of Hp1α dimerization at the periphery of the domain can further restrict protein diffusion, depletion of HP1α is not sufficient to alleviate the heterochromatin barrier and that other mechanisms are in place. This is in line with the observation that the accessibility of mouse heterochromatin to an inert protein in SUV39 KO MEFs is not increased compared with wild-type cells, suggesting that chromocenters remain a barrier to protein diffusion even in the absence of the main heterochromatin proteins (Erdel et al., 2020). In further agreement, only tethering of the strong transcriptional activator VPR (Erdel et al., 2020)—and not VP64 (Tsouroula et al., 2016)—which is sufficient to increase protein diffusion at mouse heterochromatin and to dissolve the DAPI-dense chromocenters, led to the recruitment of both RAD51 and BRCA1 within the core domain. In human cells, on the other hand, depletion of HP1 is sufficient to increase the diffusion of an inert protein in the human heterochromatin and the recruitment of BRCA1 in G1 phase of the cell cycle where it is predominantly peripheral. These results are consistent with a model (Figure S6) in which the physical organization of the different heterochromatic regions *per se* dictates the spatial activation of DNA repair pathways. In the case of non-clustered repetitive elements, compaction proteins such as HP1 can regulate accessibility of the domain. In contrast, clustering of heterochromatic repeats leads to the formation of a more inaccessible domain, which comprises several protection layers to prevent de-compaction, protein diffusion (accessibility), and illegitimate recombination.

Another scenario is that HP1 dimerization is merely a result of the DSB relocation, and when the broken chromatin is relocating from the core domain to the periphery, the HP1α molecules from the internal part dimerize with the ones at the periphery, inducing further local condensation. It was previously reported that HP1 regulates DNA end resection (Soria and Almouzni, 2013). It is, therefore, possible that the lack of HP1 reduces DNA end resection and subsequently the DNA end resection-dependent break relocation leading to the observed reduction of RAD51 recruitment at the periphery of mouse pericentric heterochromatin.

Among other nuclear compartments, mouse chromocenters have been shown to behave as membrane-less organelles through liquid-liquid phase separation (LLPS) (Banani et al., 2017; Hinde et al., 2015; Larson et al., 2017; Strom et al., 2017). Since HP1α forms liquid droplets, it is tempting to speculate that increased HP1 dimerization after DNA damage dictates the positioning of HR factors through phase separation. Another possible explanation is that RAD51 foci have themselves LLPS properties, as has been reported for 53BP1 and RAD52 DNA repair foci (Kilic et al., 2019; Oshidari et al., 2020; Pessina et al., 2019), and potential differences in the liquid properties of RAD51 and chromocenters do not allow their proper mixing.

Another model proposes that chromocenters exert hallmarks of collapsed chromatin globules (Erdel et al., 2020) rather than liquid droplets generated by heterochromatin-specific bridging interactions and the intrinsic property of major satellite repeats to self-associate. HP1α dimerization might be enhancing chromatin bridging and stabilizing the silenced heterochromatin state, decreasing the accessibility to nucleoplasmic factors.

Although other studies have shown that DSB relocation from heterochromatin occurs by directed motion, and not passive diffusion, and depends entirely (Caridi et al., 2018) or partly (Marnef et al., 2019) on actin- and myosin-related processes, they do not assess whether inhibiting these pathways is sufficient to allow HR proteins to access heterochromatin or whether these mechanisms are distinct. With the models described above, we suggest that HR exclusion from mouse heterochromatin

does not require actin/myosin-related mechanisms and highlight the fact that the differences in biophysical properties of different heterochromatin structures in mammalian cells might have fundamental implications for genome integrity.

Limitations of the study

Our results demonstrate that heterochromatic repeat clustering creates a refractory environment for HR to avoid the formation of chromosomal translocations originating from illegitimate recombination between identical repeats from different chromosomes. Our hypothesis was supported by a series of experiments in which we disrupted heterochromatic repeat clustering in mouse chromocenters. Nevertheless, our study does not explore whether the artificial induction of clustering between SatIII pericentromeric repeats in human cells is sufficient to restrict the access of HR factors at the periphery of the domain. It would, therefore, be very interesting to explore whether in senescent human cells, which can exhibit DAPI-dense senescence-associated heterochromatic foci (SAHF), we can recapitulate the results obtained in mouse cells.

STAR★METHODS

Detailed methods are provided in the online version of this paper and include the following:

- KEY RESOURCES TABLE
- RESOURCE AVAILABILITY
  - Lead contact
  - Materials availability
  - Data and code availability
- EXPERIMENTAL MODEL AND SUBJECT DETAILS
  - Cell culture
  - Cell treatments
  - Cell cycle analysis
  - Transfection
- METHOD DETAILS
  - Western Blot analysis
  - RT qPCR
  - Immunofluorescence – immuno FISH and Confocal and Super-resolution Microscopy
  - Sat III PNA FISH– immunofluorescence
  - Immuno-FISH sat II
  - Metaphase spreads and Fluorescence in Situ Hybridization (FISH)
  - Plasmid construction
  - Confocal Laser Scanning Microscopy
  - Microscopy data acquisition
  - Number and brightness (NB) analysis
  - Pair correlation function (pCF) and autocorrelation function (ACF) analysis
  - Figure preparation
- QUANTIFICATION AND STATISTICAL ANALYSIS

SUPPLEMENTAL INFORMATION

Supplemental information can be found online at https://doi.org/10.1016/j.molcel.2022.03.033.

ACKNOWLEDGMENTS

We thank K. Meaburn, F. Erdel and Matthias Altmeyer for critical reading of the manuscript and members of the Soutoglou laboratory for comments; F. Erdel for the dCas9-EGF-VPR plasmid; Y.M. Yamashita for the D1-GFP plasmid; C. Vourch for the DBD-TRIM-EGFP plasmid; D. Stroud for the mKate2 plasmid; J. Jimenez Sainz for the mCherry-BRCA2 plasmid; R. Greenberg for the Flag-TRF1-Fok1 plasmid; D. Durocher for the PALB2-GFP plasmid; H. van Attikum for the mCherry-XRCC4 plasmid; S. Daujat for the MEF cell lines; A. Nussenzweig for the BRCA1 antibody; the IGBMC Imaging Center for support; and the Biological Optical Microscopy Platform, University of Melbourne, for enabling access to the Olympus FV3000 confocal laser scanning microscope. I.M. was supported by LabEX INRT and by the Foundation ARC for the research against cancer. The E.S. laboratory was supported by the European Research Council (ERC) under the European Union's Horizon 2020 Research and Innovation program (ERC-2015-COG-682939) and by ANR-10-LABX-0030-INRT, managed by the Agence Nationale de la Recherche under the program Investissements d'Avenir, labeled ANR-10-IDEX-0002-02, and the Academy of Medical Sciences (AMS). A.K. and J.L. were supported by Australian Research Council (ARC) discovery projects (DP180101387 and DP210102984). E.H. was supported by a National Health and Medical Research Council (NHMRC) career development fellowship (APP1124762) and an ARC future fellowship (FT200100401).

AUTHOR CONTRIBUTIONS

Conceptualization, E.S. and I.M.; methodology, I.M., A.K., J.L., and E.H.; investigation, I.M., J.L., A.K., E.H., and J.V.; resources, B.R.-S.-M.; writing—original draft, E.S. and I.M.; writing—review and editing, B.R.-S.-M. and E.H.; supervision, E.S. and E.H.; funding acquisition, E.S.

DECLARATION OF INTERESTS

The authors declare no competing interests.

Received: February 19, 2021
Revised: December 15, 2021
Accepted: March 28, 2022
Published: April 20, 2022

REFERENCES

Alcobia, I., Quina, A.S., Neves, H., Clode, N., and Parreira, L. (2003). The spatial organization of centromeric heterochromatin during normal human lymphopoiesis: evidence for ontogenically determined spatial patterns. Exp. Cell Res. *290*, 358–369.

Banani, S.F., Lee, H.O., Hyman, A.A., and Rosen, M.K. (2017). Biomolecular condensates: organizers of cellular biochemistry. Nat. Rev. Mol. Cell Biol. *18*, 285–298.

Bancaud, A., Huet, S., Daigle, N., Mozziconacci, J., Beaudouin, J., and Ellenberg, J. (2009). Molecular crowding affects diffusion and binding of nuclear proteins in heterochromatin and reveals the fractal organization of chromatin. EMBO J. *28*, 3785–3798.

Brändle, F., Frühbauer, B., and Jagannathan, M. (2022). Principles and functions of pericentromeric satellite DNA clustering into chromocenters. Semin. Cell Dev. Biol.

Caridi, C.P., D'Agostino, C., Ryu, T., Zapotoczny, G., Delabaere, L., Li, X., Khodaverdian, V.Y., Amaral, N., Lin, E., Rau, A.R., and Chiolo, I. (2018). Nuclear F-actin and myosins drive relocalization of heterochromatic breaks. Nature *559*, 54–60.

Cheutin, T., McNairn, A.J., Jenuwein, T., Gilbert, D.M., Singh, P.B., and Misteli, T. (2003). Maintenance of stable heterochromatin domains by dynamic HP1 binding. Science *299*, 721–725.

Chiolo, I., Minoda, A., Colmenares, S.U., Polyzos, A., Costes, S.V., and Karpen, G.H. (2011). Double-strand breaks in heterochromatin move outside

of a dynamic HP1a domain to complete recombinational repair. Cell *144*, 732–744.

Cho, N.W., Dilley, R.L., Lampson, M.A., and Greenberg, R.A. (2014). Interchromosomal homology searches drive directional ALT telomere movement and synapsis. Cell *159*, 108–121.

Clouaire, T., and Legube, G. (2019). A snapshot on the cis chromatin response to DNA double-strand breaks. Trends Genet. *35*, 330–345.

Dantuma, N.P., and van Attikum, H. (2016). Spatiotemporal regulation of post-translational modifications in the DNA damage response. EMBO J. *35*, 6–23.

Digman, M.A., Dalal, R., Horwitz, A.F., and Gratton, E. (2008). Mapping the number of molecules and brightness in the laser scanning microscope. Biophys. J. *94*, 2320–2332.

Digman, M.A., and Gratton, E. (2009). Imaging barriers to diffusion by pair correlation functions. Biophys. J. *97*, 665–673.

Digman, M.A., Wiseman, P.W., Choi, C., Horwitz, A.R., and Gratton, E. (2009). Stoichiometry of molecular complexes at adhesions in living cells. Proc. Natl. Acad. Sci. USA *106*, 2170–2175.

Engler, C., Gruetzner, R., Kandzia, R., and Marillonnet, S. (2009). Golden gate shuffling: a one-pot DNA shuffling method based on type IIs restriction enzymes. PLoS One *4*, e5553.

Erdel, F., Rademacher, A., Vlijm, R., Tünnermann, J., Frank, L., Weinmann, R., Schweigert, E., Yserentant, K., Hummert, J., Bauer, C., et al. (2020). Mouse heterochromatin adopts digital compaction states without showing hallmarks of HP1-driven liquid-liquid phase separation. Mol. Cell *78*, 236–249.e7.

Fradet-Turcotte, A., Canny, M.D., Escribano-Díaz, C., Orthwein, A., Leung, C.C., Huang, H., Landry, M.C., Kitevski-LeBlanc, J., Noordermeer, S.M., Sicheri, F., and Durocher, D. (2013). 53BP1 is a reader of the DNA-damage-induced H2A Lys 15 ubiquitin mark. Nature *499*, 50–54.

Guenatri, M., Bailly, D., Maison, C., and Almouzni, G. (2004). Mouse centric and pericentric satellite repeats form distinct functional heterochromatin. J. Cell Biol. *166*, 493–505.

Hansen, R.K., Mund, A., Poulsen, S.L., Sandoval, M., Klement, K., Tsouroula, K., Tollenaere, M.A., Räschle, M., Soria, R., Offermanns, S., et al. (2016). SCAI promotes DNA double-strand break repair in distinct chromosomal contexts. Nat. Cell Biol. *18*, 1357–1366.

Harding, S.M., Boiarsky, J.A., and Greenberg, R.A. (2015). ATM dependent silencing links nucleolar chromatin reorganization to DNA damage recognition. Cell Rep. *13*, 251–259.

Hinde, E., Cardarelli, F., Digman, M.A., and Gratton, E. (2010). In vivo pair correlation analysis of EGFP intranuclear diffusion reveals DNA-dependent molecular flow. Proc. Natl. Acad. Sci. U. S. A. *107*, 16560–16565.

Hinde, E., Cardarelli, F., and Gratton, E. (2015). Spatiotemporal regulation of heterochromatin protein 1-alpha oligomerization and dynamics in live cells. Sci. Rep. *5*, 12001.

Hinde, E., Kong, X., Yokomori, K., and Gratton, E. (2014). Chromatin dynamics during DNA repair revealed by pair correlation analysis of molecular flow in the nucleus. Biophys. J. *107*, 55–65.

Houlard, M., Berlivet, S., Probst, A.V., Quivy, J.P., Héry, P., Almouzni, G., and Gérard, M. (2006). CAF-1 is essential for heterochromatin organization in pluripotent embryonic cells. PLOS Genet. *2*, e181.

Isono, M., Niimi, A., Oike, T., Hagiwara, Y., Sato, H., Sekine, R., Yoshida, Y., Isobe, S.Y., Obuse, C., Nishi, R., et al. (2017). BRCA1 directs the repair pathway to homologous recombination by promoting 53BP1 dephosphorylation. Cell Rep. *18*, 520–532.

Jagannathan, M., Cummings, R., and Yamashita, Y.M. (2018). A conserved function for pericentromeric satellite DNA. eLife *7*, e34122.

Jakob, B., Splinter, J., Conrad, S., Voss, K.O., Zink, D., Durante, M., Löbrich, M., and Taucher-Scholz, G. (2011). DNA double-strand breaks in heterochromatin elicit fast repair protein recruitment, histone H2AX phosphorylation and relocation to euchromatin. Nucleic Acids Res. *39*, 6489–6499.

Jensen, R.B., Carreira, A., and Kowalczykowski, S.C. (2010). Purified human BRCA2 stimulates RAD51-mediated recombination. Nature *467*, 678–683.

Jolly, C., Konecny, L., Grady, D.L., Kutskova, Y.A., Cotto, J.J., Morimoto, R.I., and Vourc'h, C. (2002). In vivo binding of active heat shock transcription factor 1 to human chromosome 9 heterochromatin during stress. J. Cell Biol. *156*, 775–781.

Kalousi, A., Hoffbeck, A.S., Selemenakis, P.N., Pinder, J., Savage, K.I., Khanna, K.K., Brino, L., Dellaire, G., Gorgoulis, V.G., and Soutoglou, E. (2015). The nuclear oncogene SET controls DNA repair by KAP1 and HP1 retention to chromatin. Cell Rep. *11*, 149–163.

Kilic, S., Lezaja, A., Gatti, M., Bianco, E., Michelena, J., Imhof, R., and Altmeyer, M. (2019). Phase separation of 53BP1 determines liquid-like behavior of DNA repair compartments. EMBO J. *38*, e101379.

Lambert, S., Mizuno, K., Blaisonneau, J., Martineau, S., Chanet, R., Fréon, K., Murray, J.M., Carr, A.M., and Baldacci, G. (2010). Homologous recombination restarts blocked replication forks at the expense of genome rearrangements by template exchange. Mol. Cell *39*, 346–359.

Larsen, D.H., Hari, F., Clapperton, J.A., Gwerder, M., Gutsche, K., Altmeyer, M., Jungmichel, S., Toledo, L.I., Fink, D., Rask, M.B., et al. (2014). The NBS1-Treacle complex controls ribosomal RNA transcription in response to DNA damage. Nat. Cell Biol. *16*, 792–803.

Larson, A.G., Elnatan, D., Keenen, M.M., Trnka, M.J., Johnston, J.B., Burlingame, A.L., Agard, D.A., Redding, S., and Narlikar, G.J. (2017). Liquid droplet formation by HP1alpha suggests a role for phase separation in heterochromatin. Nature *547*, 236–240.

Marmorstein, L.Y., Kinev, A.V., Chan, G.K., Bochar, D.A., Beniya, H., Epstein, J.A., Yen, T.J., and Shiekhattar, R. (2001). A human BRCA2 complex containing a structural DNA binding component influences cell cycle progression. Cell *104*, 247–257.

Marnef, A., Finoux, A.L., Arnould, C., Guillou, E., Daburon, V., Rocher, V., Mangeat, T., Mangeot, P.E., Ricci, E.P., and Legube, G. (2019). A cohesin/HUSH- and LINC-dependent pathway controls ribosomal DNA double-strand break repair. Genes Dev. *33*, 1175–1190.

Mitrentsi, I., and Soutoglou, E. (2021). CRISPR/Cas9-induced breaks in heterochromatin, visualized by immunofluorescence. Methods Mol. Biol. *2153*, 439–445.

Mitrentsi, I., Yilmaz, D., and Soutoglou, E. (2020). How to maintain the genome in nuclear space. Curr. Opin. Cell Biol. *64*, 58–66.

Norwood, L.E., Moss, T.J., Margaryan, N.V., Cook, S.L., Wright, L., Seftor, E.A., Hendrix, M.J., Kirschmann, D.A., and Wallrath, L.L. (2006). A requirement for dimerization of HP1Hsalpha in suppression of breast cancer invasion. J. Biol. Chem. *281*, 18668–18676.

Orthwein, A., Noordermeer, S.M., Wilson, M.D., Landry, S., Enchev, R.I., Sherker, A., Munro, M., Pinder, J., Salsman, J., Dellaire, G., et al. (2015). A mechanism for the suppression of homologous recombination in G1 cells. Nature *528*, 422–426.

Oshidari, R., Huang, R., Medghalchi, M., Tse, E.Y.W., Ashgriz, N., Lee, H.O., Wyatt, H., and Mekhail, K. (2020). DNA repair by Rad52 liquid droplets. Nat. Commun. *11*, 695.

Pankotai, T., Bonhomme, C., Chen, D., and Soutoglou, E. (2012). DNAPKcs-dependent arrest of RNA polymerase II transcription in the presence of DNA breaks. Nat. Struct. Mol. Biol. *19*, 276–282.

Pessina, F., Giavazzi, F., Yin, Y., Gioia, U., Vitelli, V., Galbiati, A., Barozzi, S., Garre, M., Oldani, A., Flaus, A., et al. (2019). Functional transcription promoters at DNA double-strand breaks mediate RNA-driven phase separation of damage-response factors. Nat. Cell Biol. *21*, 1286–1299.

Qian, H., and Elson, E.L. (1990). Distribution of molecular aggregation by analysis of fluctuation moments. Proc. Natl. Acad. Sci. USA *87*, 5479–5483.

Ryu, T., Spatola, B., Delabaere, L., Bowlin, K., Hopp, H., Kunitake, R., Karpen, G.H., and Chiolo, I. (2015). Heterochromatic breaks move to the nuclear periphery to continue recombinational repair. Nat. Cell Biol. *17*, 1401–1411.

Schrank, B.R., Aparicio, T., Li, Y., Chang, W., Chait, B.T., Gundersen, G.G., Gottesman, M.E., and Gautier, J. (2018). Nuclear ARP2/3 drives DNA break clustering for homology-directed repair. Nature *559*, 61–66.

Soria, G., and Almouzni, G. (2013). Differential contribution of HP1 proteins to DNA end resection and homology-directed repair. Cell Cycle *12*, 422–429.

Strom, A.R., Emelyanov, A.V., Mir, M., Fyodorov, D.V., Darzacq, X., and Karpen, G.H. (2017). Phase separation drives heterochromatin domain formation. Nature *547*, 241–245.

Swanson, E.C., Manning, B., Zhang, H., and Lawrence, J.B. (2013). Higher-order unfolding of satellite heterochromatin is a consistent and early event in cell senescence. J. Cell Biol. *203*, 929–942.

Tang, J., Cho, N.W., Cui, G., Manion, E.M., Shanbhag, N.M., Botuyan, M.V., Mer, G., and Greenberg, R.A. (2013). Acetylation limits 53BP1 association with damaged chromatin to promote homologous recombination. Nat. Struct. Mol. Biol. *20*, 317–325.

Tsouroula, K., Furst, A., Rogier, M., Heyer, V., Maglott-Roth, A., Ferrand, A., Reina-San-Martin, B., and Soutoglou, E. (2016). Temporal and spatial uncoupling of DNA double strand break repair pathways within mammalian heterochromatin. Mol. Cell *63*, 293–305.

Umkehrer, C., Holstein, F., Formenti, L., Jude, J., Froussios, K., Neumann, T., Cronin, S.M., Haas, L., Lipp, J.J., Burkard, T.R., et al. (2021). Isolating live cell clones from barcoded populations using CRISPRa-inducible reporters. Nat. Biotechnol. *39*, 174–178.

van Sluis, M., and McStay, B. (2015). A localized nucleolar DNA damage response facilitates recruitment of the homology-directed repair machinery independent of cell cycle stage. Genes Dev. *29*, 1151–1163.

Vourc’h, C., and Biamonti, G. (2011). Transcription of satellite DNAs in mammals. Prog. Mol. Subcell. Biol. *51*, 95–118.

Warmerdam, D.O., van den Berg, J., and Medema, R.H. (2016). Breaks in the 45S rDNA lead to recombination-mediated loss of repeats. Cell Rep. *14*, 2519–2527.

Weierich, C., Brero, A., Stein, S., von Hase, J., Cremer, C., Cremer, T., and Solovei, I. (2003). Three-dimensional arrangements of centromeres and telomeres in nuclei of human and murine lymphocytes. Chromosome Res. *11*, 485–502.

Wiblin, A.E., Cui, W., Clark, A.J., and Bickmore, W.A. (2005). Distinctive nuclear organisation of centromeres and regions involved in pluripotency in human embryonic stem cells. J. Cell Sci. *118*, 3861–3868.

STAR★METHODS

KEY RESOURCES TABLE

REAGENT or RESOURCE	SOURCE	IDENTIFIER
Antibodies		
g-H2AX (H2AX S139), 1:1000 (IF&WB)	Abcam	Cat# ab22551; RRID: AB_447150
53BP1, 1:1000 (IF)	Novus Biologicals	Cat# 100-304; RRID: AB_350221
pATM (S1981), 1:500 (IF), 1:1000 (WB)	Abcam	Cat# ab81292; RRID: AB_1640207
KAP1, 1:1000 (IF & WB)	Euromedex	Cat# 1TB1A9
MDC1, 1:1000 (IF)	Made in IGBMC	N/A
RAD51, 1:100 (IF)	Calbiochem	Cat# PC130; RRID: AB_2238184
RPA32, 1:250 (IF)	Novus Biologicals	Cat# 600-565; RRID: AB_526630
CREST, 1:500 (IF)	Antibodies Incorporated	Cat# 15-235-F; RRID: AB_2797147
mouse CENP-A, 1:500 (IF)	Cell Signaling Technology	Cat# 2048; RRID: AB_1147629
BRCA1 (for mouse cells), 1:200 (IF)	Gift from Dr. Andre Nussenzweig	N/A
BRCA1 (for human cells), 1:100 (IF)	Santa Cruz	Cat# sc-642; RRID: AB_630944
RIF1 (N20), 1:100 (IF)	Santa Cruz	Cat# sc55979; RRID: AB_2126818
GAPDH, 1:5000 (WB)	Millipore	Cat# MAB374; RRID: AB_2107445
Hp1 α, 1:500 (IF & WB)	Euromedex	Cat# 2HP1H5
Hp1 β, 1:1000 (IF & WB)	Euromedex	Cat# 1MOD1A9
Hp1 γ, 1:3000 (IF & WB)	Euromedex	Cat# 2MOD1G6
ARP2, 1:1000 (WB)	Abcam	Cat# Ab47654; RRID: AB_1139848
ARP3, 1:1000 (WB)	Abcam	Cat# Ab151289
PML, 1:100 (IF)	Santa Cruz	Cat# sc-966; RRID: AB_628162
Flag, 1:500 (IF)	Sigma	Cat# 088K6019
H3K9me3, 1:500 (IF)	Abcam	Cat# ab8898; RRID: AB_306848
Cas9, 1: 1000 (WB)	Diagenode	Cat# C15200229; RRID: AB_2889848
Chemicals, peptides, and recombinant proteins		
Cdk1 inhibitor IV RO-3306	Calbiochem	Cat# 217699
Thymidine	Sigma	Cat# T1895
Neocarnizostatin (NCS)	Sigma	Cat# N9162-100
Colcemid	Fisher Scientific	Cat# 15210040
FISH probes sequences, see Table S1	See Table S1	See Table S1
Experimental models: Cell lines		
U2OS	ATCC	Cat# HTB-96
HeLa	P. Gleeson, University of Melbourne, Australia.	N/A
NIH-3T3	ATCC	CRL-1658
hTERT RPE1	ATCC	CRL-4000
MEFs (WT and SUV39 dKO)	S. Daujat, ESBS, Illkirch, France	N/A
ES-E14TG2a (E14)	ATCC	CRL-1821
Oligonucleotides		
Scramble	Dharmacon	D-001810-01
Mouse Hp1α (Cbx5)	Dharmacon	L-040799-01
Mouse Hp1β (Cbx1)	Dharmacon	L-060281-01
Mouse Hp1γ (Cbx3)	Dharmacon	L-044218-01
Human Hp1α	Dharmacon	L-004296-00
Human Hp1β	Dharmacon	L-009716-00

(*Continued on next page*)

Continued

REAGENT or RESOURCE	SOURCE	IDENTIFIER
Human Hp1γ	Dharmacon	L-010033-00
Mouse siARP2	Dharmacon	L-053600-01
Mouse siARP3	Dharmacon	L-046642-01
Mouse siUNC45	Dharmacon	L-051936-01
Mouse Caf1 p150, see Table S2 for sequence	Sigma (custom)	N/A
Sequences of primer pairs used for RTqPCR in this study, see Table S2	This paper	N/A
Sequence of CRISPR-Cas9 guide-RNAs (gRNAs), see Table S3	This paper	N/A
Recombinant DNA		
pCX-5	Tsouroula et al. (2016)	N/A
pCX-15	Tsouroula et al. (2016)	N/A
pX-86	Tsouroula et al. (2016)	N/A
pG-56	Tsouroula et al. (2016)	N/A
pX-473 (U6p-gRNA (satIII #347)-SV40p-Cas9-EGFP-PuroR-pA)	This paper	N/A
pX-475 (U6p-gRNA (satIII #349)-SV40p-Cas9-EGFP-PuroR-pA)	This paper	N/A
pX-479 (U6p-gRNA (satIII #349)-SV40p-dCas9-EGFP-PuroR-pA)	This paper	N/A
pX-789 (U6p-gRNA(Ma-Sat#3)-SV40p-EGFP-dCas9-hRad51-PuroR-pA)	This paper	N/A
satII-Cas9 (U6p-gRNA (satII)-SV40p-Cas9-EGFP-PuroR-pA)	This paper	N/A
EGFP-dCas9-BRC3 (U6p-gRNA(Ma-Sat#3)-SV40p-EGFP-dCas9-BRC3-PuroR-pA)	This paper	N/A
EGFP-dCas9-VPR	Fabian Erdel lab, CBI, Toulouse, France (Erdel et al., 2020)	N/A
D1-GFP	Y.M Yamashita lab, MIT, USA (Jagannathan et al., 2018)	N/A
DBD-TRIM -EGFP	C.Vourc'h lab, UGA, Grenoble, France (Jolly et al., 2002)	N/A
DBD-TRIM -mCherry	This paper	N/A
mKate2	D. Stroud, University of Melbourne, Australia	N/A
RFP657-HP1α	This paper	N/A
eGFP-53BP1	D.Durocher lab, Lunenfeld-Tanenbaum Research Institute, Canada (Fradet-Turcotte et al., 2013)	Addgene, 60813
Major Satellite repeats	(Tsouroula et al., 2016)	N/A
mCherry BRCA2	J. Jimenez Sainz lab, Yale School of Medicine, USA	N/A
EGFP-HP1αI165E	L.L. Wallrath lab, University of Iowa, USA (Norwood et al., 2006)	N/A
EGFP-HP1α	Tom Misteli lab, NIH, USA (Cheutin et al., 2003)	Addgene, 17652
Flag TRF1-FokI	R. Greenberg lab, Perelman School of Medicine, USA (Tang et al., 2013)	N/A
dCas9-VPR-mCherry	Anna Obenauf lab, IMP, Vienna, Austria (Umkehrer et al., 2021)	Addgene, 154193

(*Continued on next page*)

Continued

REAGENT or RESOURCE	SOURCE	IDENTIFIER
PALB2-GFP	D.Durocher lab, Lunenfeld-Tanenbaum Research Institute, Canada (Orthwein et al., 2015)	N/A
XRCC4 mCherry	H.Van Attikum lab, Leiden University Medical Center	N/A
GFP-Hp1α	(Kalousi et al., 2015)	N/A
Software and algorithms		
Fiji/ImageJ	National Institutes of Health	https://imagej.nih.gov/ij/
CellProfiler 4.1.3, Cell profiler image analysis Software	Carpenter Lab at the Broad Institute of Harvard and MIT	https://cellprofiler.org/releases
GraphPad Prism 9.00 for Windows, data visualization and statistics	GraphPad Software, LLC	https://www.graphpad.com/
Snapgene	Insightful Science	https://www.snapgene.com/snapgene-viewer/

RESOURCE AVAILABILITY

Lead contact

Further information and requests for resources and reagents should be directed to and will be fulfilled by the lead contact Evi Soutoglou (e.soutoglou@sussex.ac.uk).

Materials availability

The plasmids generated in this study are available from the Lead Contact upon request.

Data and code availability

- This paper does not report unprocessed data.
- This paper does not report original code.
- Any additional information required to reanalyze the data reported in this paper is available from the lead contact upon request.

EXPERIMENTAL MODEL AND SUBJECT DETAILS

Cell culture

NIH-3T3 mouse fibroblasts were maintained in high glucose DMEM supplemented with 10% newborn calf serum and gentamycin (40 mg/ml). U2OS human osteosarcoma cells were maintained in low glucose DMEM supplemented with 10% Fetal calf serum and gentamycin (40 mg/ml). HeLa (human cervical carcinoma) cells were maintained in high glucose DMEM supplemented with 10% fetal calf serum and gentamycin (40 mg/ml). RPE1 (human, retina epithelial) cells were maintained in DMEM/F12 with GLUTAMAX-I, which was supplemented with 10% Fetal calf serum and gentamycin (40 mg/ml). E14 cells (mouse embryonic stem cells) were maintained in high glucose DMEM supplemented with 10% Fetal Calf Serum, MEM Non-essential amino acids, L- Glutamine (200mM), β- mercaptoethanol (50 mM), Leukemia Inhibiting Factor and Penicilin/Streptomycin (40 mg/ml).

Cell treatments

For fixed cells experiments, cells were synchronized in the G2 phase of the cell cycle with the Cdk1 inhibitor IV RO-3306 (217699; Calbiochem; 10 μM). For NIH-3T3 cells, the inhibitor was added 8h before transfection, with the cells fixed 16h after transfection. For the human cell lines (U2OS, HeLa, RPE1), the Cdk1 inhibitor was added 16 h before transfection and cells were fixed 8 afterward transfection, thus, in both cases leading to a total of 24h treatment. Cells were arrested in G1/S phase of the cell cycle with a double-thymidine (T1895; Sigma) block: 18h thymidine treatment (2 mM), 9h release, 16h thymidine treatment (2 mM). Cells were then transfected for 8h prior to fixation. Cell-cycle arrest was confirmed by flow cytometry. Neocarzinostatin (NCS; N9162-100 UG; Sigma) was added (100 or 200 ng/ml), 15 min later medium was replaced, and cells were harvested for Western blot analysis 1 h later.

For live-cell microscopy experiments, the NIH-3T3 and HeLa cells were plated onto 35 mm glass bottom dishes (FluoroDish FD35-100) and were synchronized into G1 and G2 according to the same protocol listed for the fixed-cell experiments. For both G1 and G2 cells, live-cell microscopy experiments were performed 8 hours post-transfection.

Cell cycle analysis

EdU incorporation and staining was performed according to the manufacturer's instructions (Click-iT EdU Flow Cytometry Assay Kit, Invitrogen). Then, cells were treated with RNAse A (100 μg/ml) and stained with propidium iodide (40 μg/ml) for 30 min at 37°C. Flow cytometry was performed on a FACSCalibur (Becton-Dickinson) and analyzed with FlowJo (TreeStar).

Transfection

Transient transfections were performed with Lipofectamine 2000/3000 (Invitrogen Life Technologies) for NIH-3T3 and E14 cells and JetPei (Polyplus transfection) for U2OS, HeLa and RPE1 cells according to the manufacturer's instructions. siRNA transfections were performed with Lipofectamine RNAi max, according to the manufacturer's instructions. For siRNAs used see key resources table and Table S2.

METHOD DETAILS

Western Blot analysis

Proteins were fractionated by SDS-PAGE and transferred to Protran Nitrocellulose membranes (Sigma Aldrich) and blotted with the antibodies listed in key resources table.

RT qPCR

RNA extraction (QIAGEN, 74106) was performed according to the manufacturer's instructions. RT qPCR was performed in triplicate using SyberGreen (QIAGEN, 204143) and a LightCycler 480 (Roche, 05015278001) as previously described (Pankotai et al., 2012). Relative quantification of transcript quantities were calculated from standard after normalizing it to GAPDH mRNA (for primers see Table S2).

Immunofluorescence – immuno FISH and Confocal and Super-resolution Microscopy

Cells were cultured on coverslips and pre-extracted in 0.1% Triton/1X PBS for 30 s prior to fixation in 4% paraformaldehyde (PFA)/1X PBS for 10 min on ice. After a second fixation step of 4% PFA/1X PBS for 10 min at room temperature, cells were permeabilized in 0.5% Triton/1X PBS for 10 min, blocked in 5% BSA/1X PBS/0.1% Tween for 1h and incubated with primary antibody (in 1% BSA/1X PBS/0.1%Tween) for 1 hr (see key resources table for antibodies) and secondary antibody (Alexa Fluor, Thermofisher) for 1 hr (in 1% BSA/1X PBS/0.1%Tween). Cells were counterstained with DAPI (1 mg/ml) and mounted on slides (Mitrentsi and Soutoglou, 2021). For EdU incorporation, the Click-iT EdU Alexa Fluor 488 Imaging Kit (Invitrogen) was used. For immuno-FISH, the same protocol was used, except the secondary antibody incubation was followed by a post-fixation in 4% PFA/PBS for 10 min. Cells were washed in 2X SSC for 45 minutes, at 72 °C. This was followed by sequential washes with 70% and absolute ethanol. The coverslips were dried at room temperature, incubated with 0.1N NaOH for 10 minutes and washed with 2X SSC. Then they were washed with 70% ethanol and absolute ethanol. After drying, cells were hybridized with DNA probe (major satellite repeats) for 30sec at 85°C and incubated overnight at 37°C.

The immuno-FISH probe was prepared by nick translation from the major satellite repeats containing plasmid (see key resources table). Probe DNA (0.3 mg) was mixed with 9 μg of ssDNA and precipitated with 2.50 vol of ethanol and 1/10 vol of 2.5M sodium acetate for 1h at -80°C. After 20 min of centrifugation, the supernatant was discarded, and the pellet was washed with70%ethanol and centrifuged again for 5min.The supernatant was discarded, and the pellet was dried. The pellet was resuspended in hybridization solution (50%formamide, 2X SSC, 10% dextran sulfate) (20 μl per coverslip) by vortexing for 1h. The probe was denaturated for 5 min at 90°C and pre annealed for at least 30 min at 37°C before hybridization with cells.

The day after hybridization, coverslips were washed twice in 2X SSC for 20 minutes at 42°C. Finally, the cells were incubated with the secondary antibody (AlexaFluor Thermofisher) and Neutravidin-Texas Red (Thermofisher). Cells were counterstained with DAPI (1 mg/ml) and mounted on slides. Cells were observed on a confocal laser scanning microscope (TCS SP8; Leica) using a 63x objective, an OMX BLAZE 3D-structured illumination, super-resolution microscope or on a NIKON inverted confocal spinning disk microscope with a GATACA Live SR module.

Sat III PNA FISH– immunofluorescence

For sat III staining, FISH was performed before the immunofluorescence. More specifically, cells were fixed in 4% PFA/PBS for 10 minutes in RT, permeabilized for 10 minutes with Triton 0.5%/PBS and washed three times with PBS. Next cells were dehydrated through an ethanol series of 50%, 70% and absolute for 3 minutes each at RT and coverslips were air-dried. Hybridization buffer (70% formamide, 10mM Tris pH 7.2) containing 50nM of PNA sat III probe (see Table S1) was added on a slide, covered with the coverslip containing the cells and they were denatured on an 80°C hot plate for 3 minutes. This was followed by hybridization for 2h at RT. Coverslips were then washed twice in 70% formamide, 10mM Tris pH7.2 for 15 minutes, then three times in 50mMTris pH7.5/150mM NaCl/ 0.05% Tween for 5 minutes and finally twice with PBS. Subsequently, the cells were blocked in 5% BSA/1X PBS/0.1% Tween for 1h and incubated with primary antibody (in 1% BSA/1X PBS-0.1%Tween) for 1 hr (see key resources table for antibodies) and secondary antibody for 1 hr (in 1% BSA/1X PBS-0.1%Tween). Cells were counterstained with DAPI (1 mg/ml) and mounted on slides.

Immuno-FISH sat II

Cells were permeabilized in ice-cold CSK Buffer (10 mM HEPES-KOH, pH 7.4, 300 mM sucrose, 100 mM NaCl, 3 mM MgCl2) containing 0.5% Triton X-100 for 5 min, washed in CSK Buffer for 5 min, and then fixed with 2% PFA/2% sucrose for 10 min, on ice. They were then permeabilized in cold 0.4% Triton X-100 / 1X PBS for 10 min on ice. Next cells were blocked in 5% BSA/1X PBS/0.1% Tween for 1h and incubated with primary antibody (in 1% BSA/1X PBS/0.1%Tween) for 1 hr (see key resources table for antibodies) and secondary antibody for 1 hr (in 1% BSA/1X PBS-0.1%Tween). This was followed by a post-fixation with 2% PFA/2% Sucrose for 10 min on ice and cells were incubated with 4 mg/mL RNase A in 2X SSC for 30 min, at 37 °C. Cells were re-permeabilized with cold 0.7% Triton X-100 / 0.1M HCl for 10 min on ice, and denatured in 2M HCl for 30 minutes at RT. After three quick rinses in cold 1X PBS, cells were incubated with Hybridization Buffer (100 ng/μL salmon sperm DNA, 5X Denhart's solution, 10% dextran sulfate, 5X SSC, 50% formamide) containing 10 ng/μL sat II FISH probe (See Table S1) overnight in a dark and humid chamber, at 37°C. The next day, coverslips were washed three times in 2X SSC/0.1% Tween for 5 min at RT and blocked with 5% BSA/1X PBS/0.1% Tween for 30 min at RT. Subsequently, cells were incubated with Streptavidin and secondary antibody for 1 hr (in 1% BSA/1X PBS/0.1% Tween). Finally, Cells were counterstained with DAPI (1 mg/ml) and mounted on slides.

Metaphase spreads and Fluorescence in Situ Hybridization (FISH)

Colcemid was added in the cells (0.02 μg/ml, 15210040; Fisher Scientific) for 4 h. Cells were trypsinized, harvested, and the resulting cell pellet was resuspended in a prewarmed 0.06M KCl solution and incubated for 30 min at 37°C. The cell pellet was fixed in an ethanol:glacial acetic acid (3:1) solution, 3 times. The following day, metaphase chromosomes were spread on wet cold glass slides, and air-dried. The spreads were fixed with 4% PFA/PBS for 4 min at 37°C, and treated with RNAse A solution (100 μg/ml in 2X SSC) for 1h at 37°C. Afterwards cells were dehydrated through an ethanol series (70%, 85%, absolute) and air-dried. DNA was denatured at 85°C for 10 minutes after the addition of 0.07μM PNA probe and/or whole chromosome 9 probe (see Table S1). This was followed by hybridization for 2h at room temperature. Slides were washed twice in SSC/0.1% Tween at 60°C for 10 min. DNA was counterstained with DAPI (1mg.ml) and mounted with a long coverslip.

Plasmid construction

Individual gRNAs (see Table S3) were cloned into a vector containing the U6 promoter followed by a gRNA scaffold. All plasmids (see key resources table) were assembled by golden gate cloning (Engler et al., 2009).

Confocal Laser Scanning Microscopy

All microscopy measurements were performed on an Olympus FV3000 laser scanning microscope coupled to an ISS A320 Fast FLIM box for fluorescence fluctuation data acquisition. A 60X water immersion objective 1.2 NA was used for all experiments and the cells were imaged at 37 °C in 5% CO2. For single channel Number and Brightness (NB) fluorescence fluctuation spectroscopy (FFS) measurements, RFP657-HP1α was excited by a solid-state laser diode operating at 633 nm and the resulting fluorescence signal was directed through a 405/488/561/633 dichroic mirror to a photomultiplier detector (H7422P-40 of Hamamatsu) fitted with a 676/29 nm bandwidth filter. For the pair correlation function (pCF) FFS measurements of mKate2 or mCherry tagged proteins, these constructs were excited by a solid-state laser diode operating at 561 nm and the resulting signal was directed through a 405/488/561 dichroic mirror to an internal GaAsP photomultiplier detector set to collect 600-700 nm. For the pair correlation function (pCF) and autocorrelation function (ACF) FFS measurements of eGFP tagged proteins, these constructs were excited by a solid-state laser diode operating at 488 nm and the resulting signaling was directed through a 405/488/561 dichroic mirror to an internal GaAsP photomultiplier detector set to collect 500-570 nm.

Microscopy data acquisition

NB FFS measurements of RFP657-HP1α involved selecting a 10.6 μm region of interest within a NIH-3T3 or Hela cell nucleus, which for a 256 x 256 pixel frame size resulted in a pixel size of 41 nm, and then acquisition of a time series of frames (n = 100) with the pixel dwell time set to 12.5 μs, which resulted in a line time of 4.313 ms and a frame time of 1.108 s. pCF FFS measurements of mKate2, PALB2-eGFP or XRCC4-mCh tagged proteins involved selecting a 5.3 μm line across the middle of a DBD-TRIM-EGFP or RFP657-HP1-RFP657 labelled foci, which for a 64 x 1 pixel line resulted in a pixel size of 83 nm, and then acquisition of a time series of lines (n = 100,000) at maximum speed (8 μs pixel dwell time / 1.624 ms line time).

Number and brightness (NB) analysis

The apparent brightness (B) of a fluorescently tagged protein is a readout of that protein's oligomeric state and this parameter can be extracted by a moment-based Number and brightness (NB) analysis of an FFS frame scan acquisition (Digman et al., 2008; Digman et al., 2009; Qian and Elson, 1990). In brief, within each pixel of a frame scan we have an intensity fluctuation that has an average intensity (first moment) and a variance (second moment), where the ratio of these two properties describes the apparent brightness (B) of the molecules that give rise to the intensity fluctuation. The true molecular brightness (ϵ) of the molecules is related to the measured B by $B = \varepsilon + 1$, where 1 is the brightness contribution of the photon counting detector. Calibration of the apparent brightness of monomeric RFP657-HP1α (I165E) ($B_{monomer} = 1.15$) enabled extrapolation of the expected apparent brightness of wild type RFP657-HP1α dimers ($B_{dimer} = 1.30$), as well as definition of brightness cursors to extract and spatially map the fraction of this

species within a given frame scan acquisition. In Figure 5A mask based on HP1α intensity was used to quantify the fraction of RFP657-HP1α dimers in the nucleoplasm versus within foci. Artefact due to cell movement or photobleaching were subtracted from acquired intensity fluctuations via use of a moving average algorithm. All brightness calculations were carried out in SimFCS from the Laboratory for Fluorescence Dynamics (www.lfd.uci.edu).

Pair correlation function (pCF) and autocorrelation function (ACF) analysis

As described in previously published papers, pCF and ACF analysis of spatially distinct fluorescence fluctuations acquired along a confocal line scan acquisition can track the evolution of protein transport as well as the number of molecules moving within the different environments that are encountered (Digman and Gratton, 2009; Hinde et al., 2010; Hinde et al., 2014). In brief, in each pixel of a line scan we have an intensity fluctuation that we can format into an intensity carpet, where the *x*-coordinate corresponds to the point along the line (pixel) and the *y*-coordinate corresponds to the time of acquisition. The carpet data format then enables both temporal cross correlation of pairs of intensity fluctuations separated by a set distance ($\delta r > 0$) along the line scan for all possible delay times (τ) by the pCF function, as well as temporal cross correlation of each intensity fluctuation along the line scan with itself ($\delta r = 0$) for all possible τ by the ACF function. The resulting pCF profile reports the characteristic times it takes a population of molecules to translocate the set distance (which in the case of Figure 4 was 480 - 640 nm since the FFS line scan measurements presented employed an 80 nm pixel size and $\delta r = 6\text{-}8$) and the resulting ACF amplitude reports the concentration gradient of this population (since at $\tau = 0$ the ACF has an amplitude inversely proportional to the number of moving molecules present). The distance range $\delta r = 6\text{-}8$ was chosen because it enabled the accessibility of the DBD-TRIM-EGFP or EGFP-HP1α or RFP657-HP1α foci toward mKate, XRCC4-mCh, and GFP-PALB2 diffusion to be tested. The ACF analysis was performed to facilitate pCF interpretation and place any detected changes in heterochromatin accessibility toward mKate diffusion within the context of changes in XRCC4 or PALB2 concentration. Artefact due to cell movement or cell bleaching were subtracted from acquired intensity fluctuations via use of a moving average algorithm. All pCF calculations were carried out in SimFCS from the Laboratory for Fluorescence Dynamics (www.lfd.uci.edu).

Figure preparation

Figures were prepared by use of Adobe Illustrator, MATLAB, and SimFCS.

QUANTIFICATION AND STATISTICAL ANALYSIS

Statistical analysis was performed by using GraphPad Prism software. For all statistical analysis, unless mentioned otherwise, were evaluated by one-way ANOVA (*p<0,05, **p<0,01, ***p<0,001, ****p<0.0001).

Molecular Cell, Volume *82*

Supplemental information

Heterochromatic repeat clustering imposes a physical barrier on homologous recombination to prevent chromosomal translocations

Ioanna Mitrentsi, Jieqiong Lou, Adèle Kerjouan, John Verigos, Bernardo Reina-San-Martin, Elizabeth Hinde, and Evi Soutoglou

SUPPLEMENTARY Figure S1 (related to Figure 1)

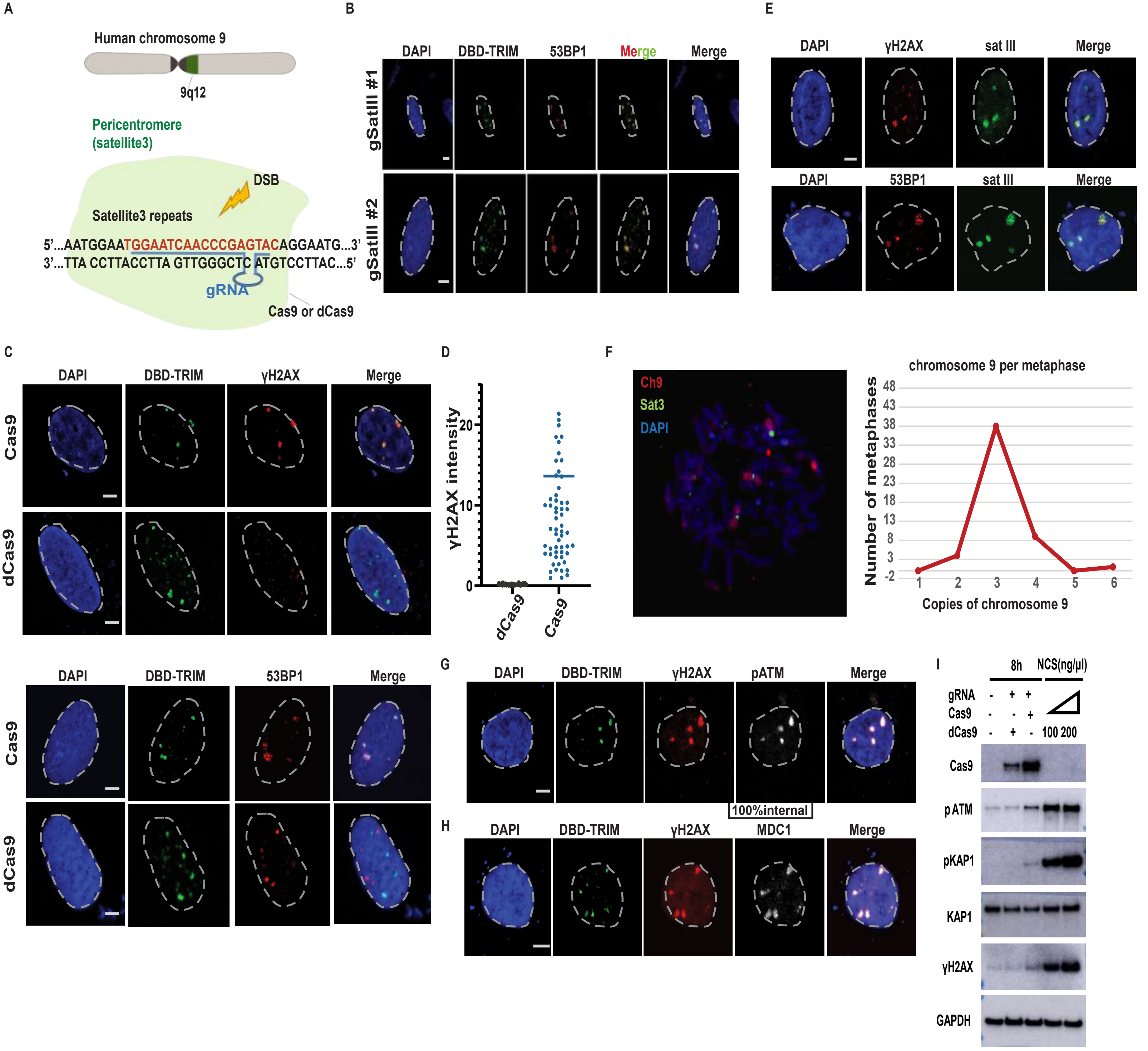

SUPPLEMENTARY Figure S2 (Related to Figure 1)

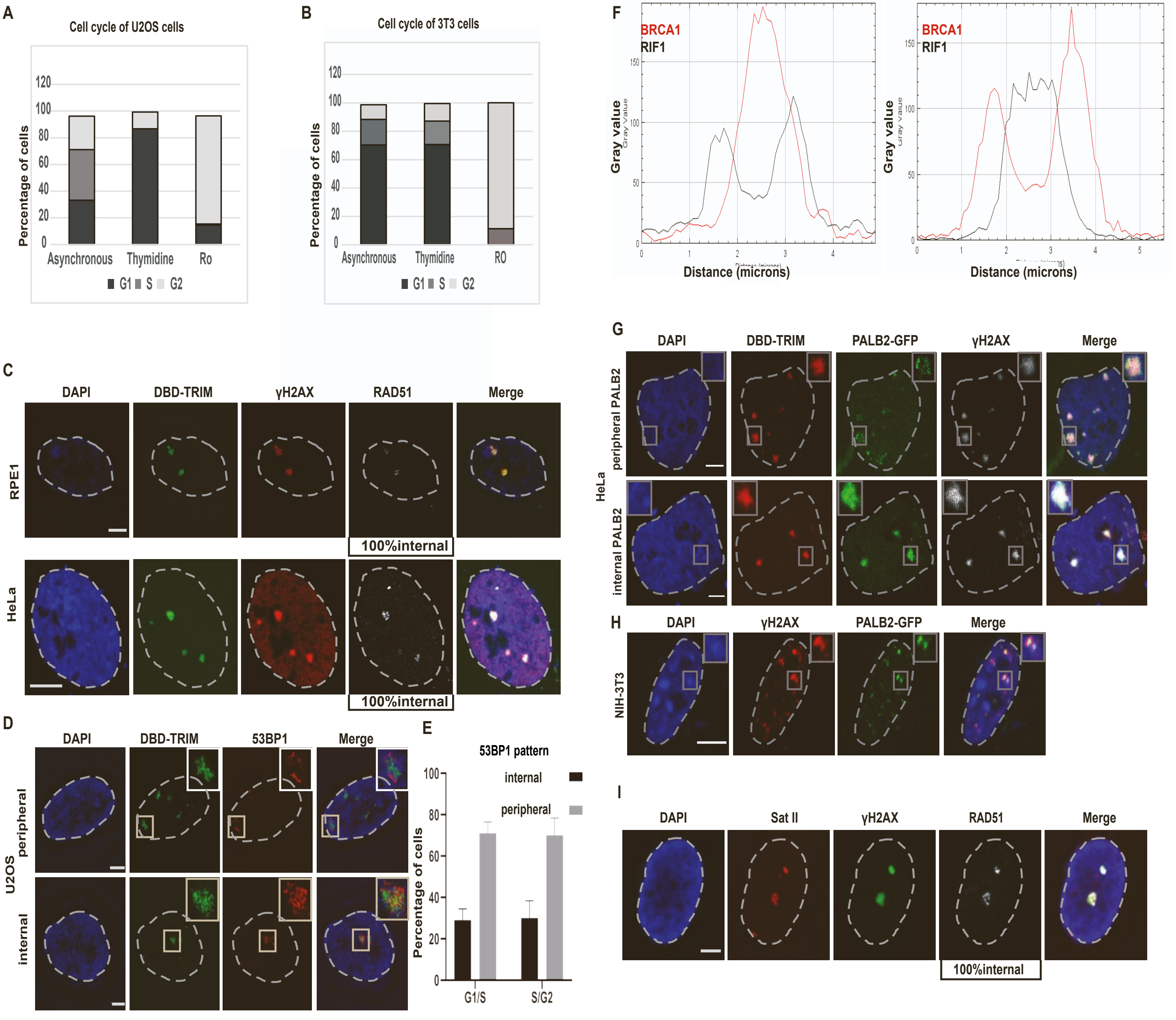

SUPPLEMENTARY Figure S3 (Related to Figure 2)

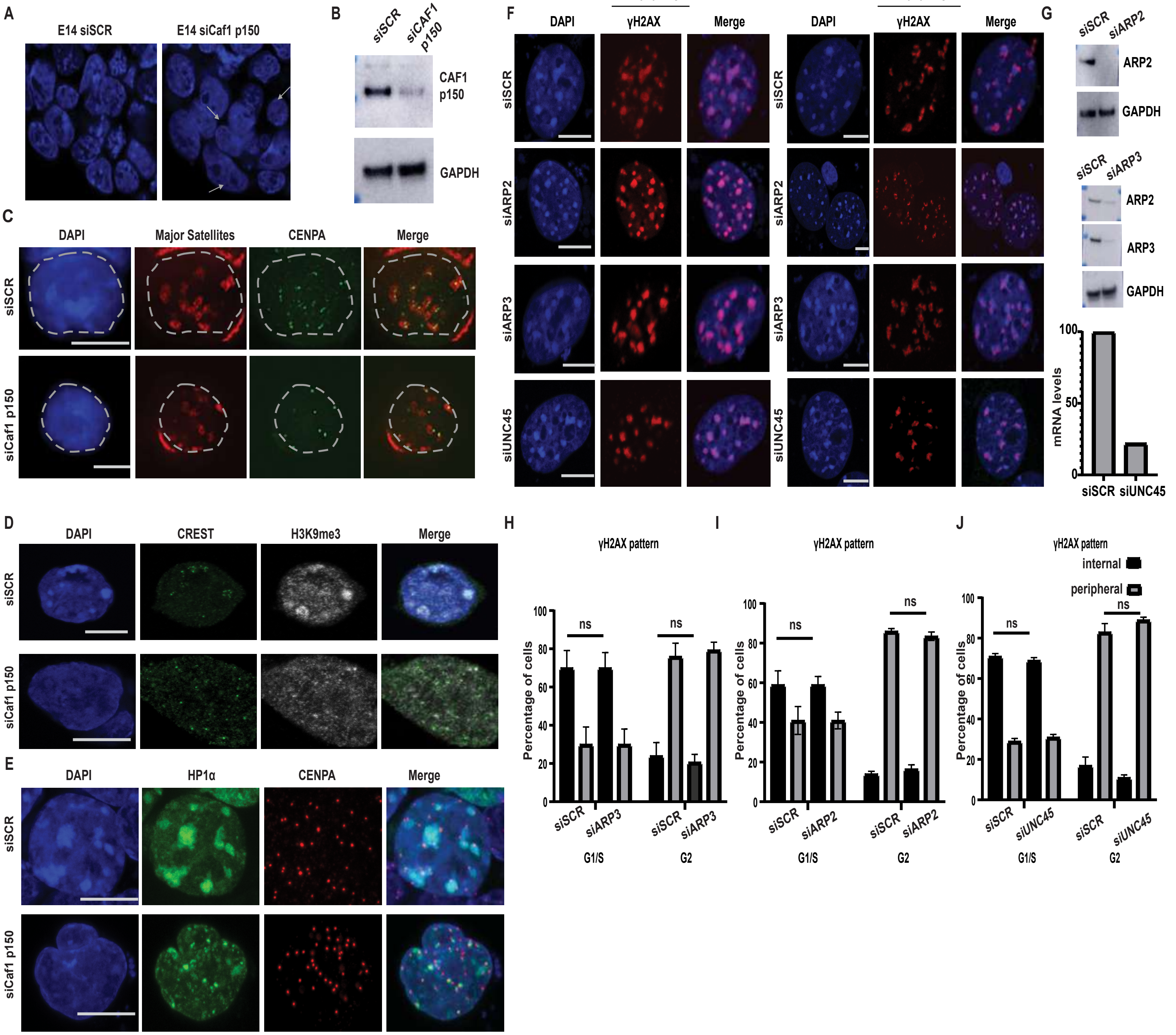

SUPPLEMENTARY Figure S4 (Related to Figure 4)

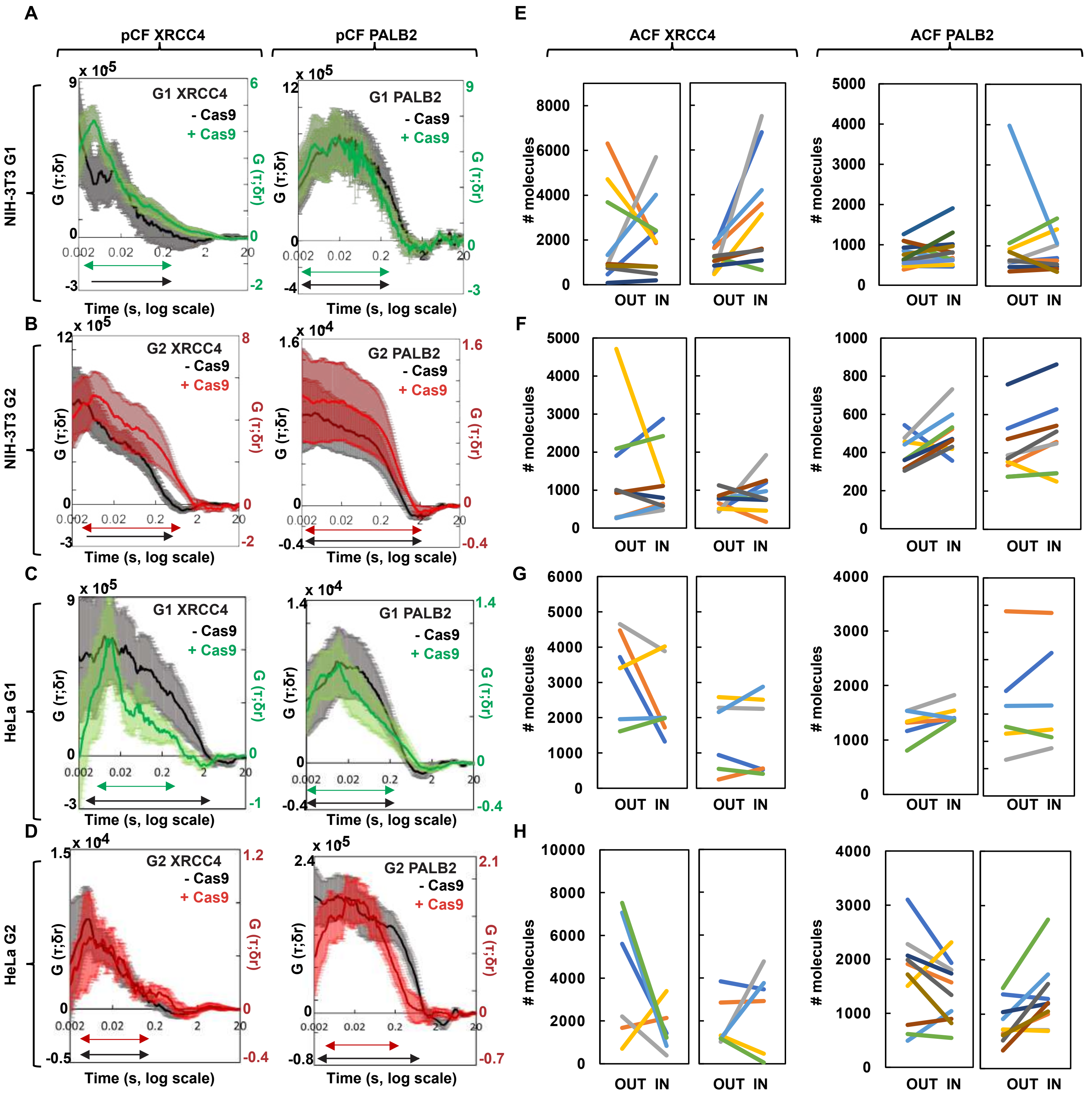

SUPPLEMENTARY Figure S5 (Related to Figure 5)

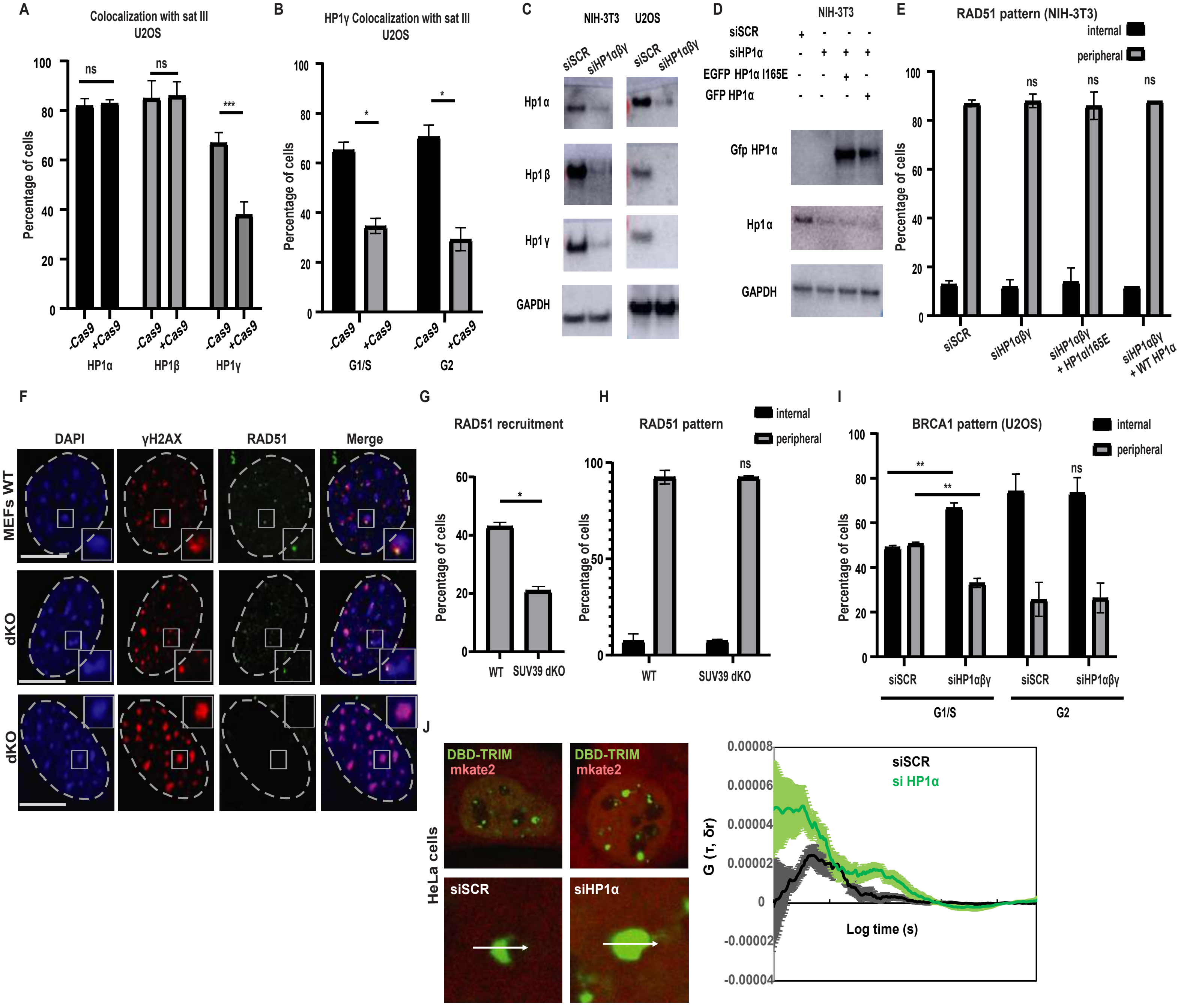

SUPPLEMENTARY Figure S6 (Related to Discussion)

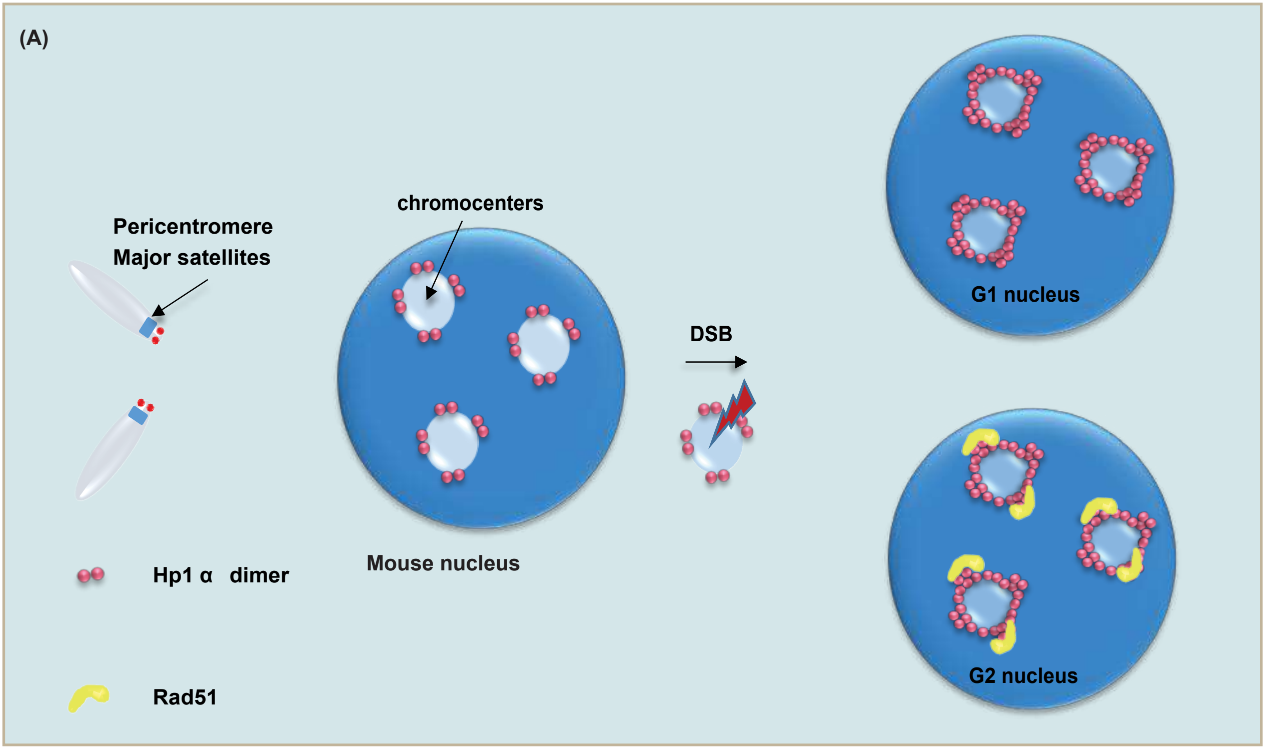

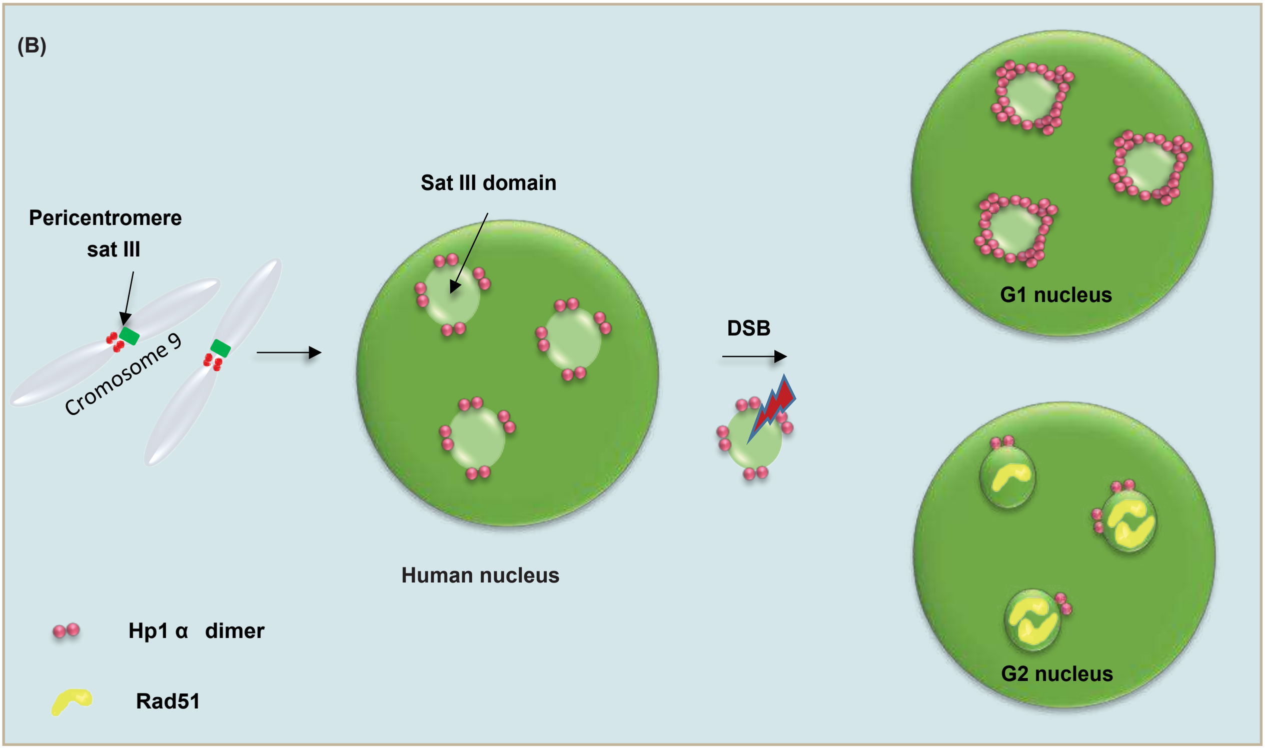

Supplementary Figure Legends

Figure S1 related to Figure1. Experimental system to induce Cas9 specific DSBs on human satIII pericentromeric regions (A) Schematic representation of the experimental system. Immunofluorescence (IF) confocal analysis of **(B)** cells co-expressing DBD-TRIM-EGFP+Cas9 + two different gRNAs specific for SatIII repeats stained with DAP1 and a 53BP1-specific antibody and **(C)** cells co-expressing DBD-TRIM-EGFP, a gRNA specific for SatIII repeats and either catalytically inactive dead Cas9 (dCas9) or Cas9 and stained with DAPI and antibodies specific for γH2AX and 53BP1. **(D)** Quantification of γH2AX intensity at sat III domains in cells co-expressing DBD-TRIM-EGFP, a gRNA specific for SatIII repeats and either dCas9 or Cas9 **(E)** IF combined with Fluorescence *in situ* Hybridization (ImmunoFISH) confocal analysis in U2OS cells expressing Cas9 + gRNA specific for the SatIII repeats stained with DAPI, a PNA-Sat III probe (green) and antibodies specific for γ-H2AX and 53BP1. **(F)** Representative confocal image of metaphase spreads after FISH with a PNA-SatIII probe (green) and chromosome paint (chromosome 9, red) and quantification of chromosome 9 copies per metaphase in U2OS cells. Data are the mean of 2 experiments (n= 50 cells). **(G-H)** IF confocal analysis of U2OS cells expressing DBD-TRIM-EGFP + Cas9 + gRNA specific for SatIII repeats stained with DAPI and antibodies specific for γ-H2AX, $pATM^{S1981}$ and MDC1. Images are representative of 3 experiments with n=50 cells. **(I)** Western blot analysis for Cas9, γ-H2AX, $pATM^{S1981}$, KAP1, $pKAP1^{S824}$ and GAPDH in protein extracts prepared from U2OS cells expressing Cas9 + gRNA specific for SatIII repeats or treated with NCS (100 ng/ml and 200 ng/ml).

Figure S2 related to Figure 1. Recruitment of HR and NHEJ factors at pericentromeric DSBs of different cell lines Cell cycle analysis by flow cytometry using propidium iodide and EdU in U2OS **(A)** and NIH-3T3 **(B)** cells treated or not with double thymidine (synchronization in G1/S) or RO-3306 (synchronization in S/G2. **(C)** IF and super resolution (upper panel) or confocal (lower panel) analysis of RPE1 (upper panel) or HeLa (lower panel) of cells expressing DBD-TRIM-EGFP+Cas9+gRNA targeting SatIII repeats stained with DAPI and antibodies specific for γ-H2AX and RAD51. **(D)** IF super resolution (3D-SIM) analysis of U2OS cells expressing DBD-TRIM-EGFP + Cas9 + gRNA targeting SatIII repeats stained with DAPI and a 53BP1-specific antibody. The upper panel represents a peripheral 53BP1 pattern and the lower internal. **(E)**Quantification of 53BP1 pattern after DSB induction in either G1/S or S/G2 synchronized U2OS cells. **(F)** Line scan analysis of BRCA1 and RIF1 spatial evolution at sat III domains on U2OS cells expressing Cas9+gRNA specific for sat III repeats. **(G)** IF confocal analysis of HeLa cells expressing DBD-TRIM-mCherry +PALB2-GFP+Cas9 + gRNA targeting SatIII repeats stained with DAPI and γH2AX specific antibody. Upper cell shows an internal PALB2 pattern whereas lower cell shows a peripheral PALB2 pattern. **(H)** IF confocal analysis of NIH-3T3 cells expressing PALB2-GFP, Cas9 + gRNA targeting major satellite repeats, stained with DAPI and γH2AX specific antibody. **(I)** ImmunoFISH confocal analysis of U2OS cells expressing Cas9 + gRNA targeting SatII repeats stained with DAPI, a SatII-specific probe, and antibodies specific for γH2AX and RAD51. Scale bars represent 5 µm. All images are representative of 3 experiments with n= 50 cells. Wherever a quantification is not provided, 100% of cells exert the phenotype.

Figure S3 related to Figure 2. Disruption of heterochromatic clustering on mouse ES cells and the role of actin/myosin mechanisms on DSB relocation (A) Confocal analysis of WT E14 cells or E14 cells depleted for Caf1 p150 (siCAF1p150; right panel) or with a non-targeting control (siSCR; left panel) stained with DAPI. The arrows indicate cells with the phenotype of disrupted clustering **(B)** Western blot analysis of CAF1 levels at WT E14 cells and E14 cells depleted of Caf1 p150. **(C)** ImmunoFISH confocal analysis of WT E14 cells (upper panel) or E14 cells depleted for Caf1 p150 (lower panel) cells expressing Cas9 + gRNA targeting major satellite repeats stained with DAPI, a major satellite-specific probe and a CENP-A specific antibody. Images are representative of 3 experiments with n = 50 cells. IF confocal analysis of WT E14 cells or E14 cells depleted for Caf1 p150, stained with DAPI and antibodies specific for CREST and H3K9me3 **(D)** or HP1α and CENPA **(E). (F)** IF confocal analysis of NIH-3T3 cells expressing Cas9 + gRNA targeting major satellites, depleted for ARP2 (siARP2), ARP3 (siARP3), UNC45 (siUNC45) or a non-targeting control (siSCR) in different stages of the cell cycle and stained with DAPI and an antibody specific for γH2AX. **(G)** siRNA efficiencies of siARP2, siARP3 (by western blot analysis) and siUNC45 (by RT-qPCR). **(H-J)** Quantification of the γH2AX pattern at chromocenters of NIH-3T3 cells expressing Cas9 + gRNA specific for the major satellite repeats, either depleted of ARP3 (siARP3) **(H)**, ARP2 (siARP2) **(I)** , or UNC45(siUNC45) **(J)** or with a non-targeting control (siSCR) in G1 or G2 phases of the cell cycle. Scale bars represent 5 μm. Data are the mean ± SD of 3 experiments with n=50 cells. For all graphs, statistical significance was determined by t-test (*p<0,05, **p<0,01, ***p<0,001).

Figure S4 related to Figure 4 pCF and ACF analysis of PALB2 and XRCC4 access on mouse versus human heterochromatin, throughout the cell cycle pCF analysis of the access of PALB2-GFP or mCherry-XRCC4 into heterochromatic DSBs and ACF analysis of the fraction of each DNA repair

factor present and bound within the indicated nuclear structures. **(A)-(B)** pCF6-8 (δr = 6-8 pixels) analysis of XRCC4 (left) versus PALB2 (right) access into NIH-3T3 in G1 **(A)** and G2 **(B)** heterochromatin in the absence (grey) versus presence (G1-green, G2-red) of DSBs (N=6-12 measurements, n>= 5cells, 2 biological replicates) and ACF (δr=0 pixels). **(C)-(D)** pCF6-8 (δr = 6-8 pixels) analysis of XRCC4 (left) versus PALB2 (right) access into HeLa in G1 **(C)** and G2 **(D)** heterochromatin in the absence (grey) versus presence (G1-green, G2-red) of DSBs (N=8-10 measurements, n>=5 cells, 2 biological replicates). **(E)-(F)** ACF analysis (δr = 0 pixels) of XRCC4 (left) versus PALB2 (right) mobility inside NIH-3T3 in G1 **(E)** and G2 **(F)** heterochromatin enables extraction of the number of moving molecules outside versus inside this nuclear structure, which underpins calculation of the ratios presented in Fig. 4J and L (N=6-12 measurements, n>= 5cells, 2 biological replicates). **(G)-(H)** ACF analysis (δr = 0 pixels) of XRCC4 (left) versus PALB2 (right) mobility inside HeLa in G1 **(G)** and G2 **(H)** heterochromatin enables extraction of the number of moving molecules outside versus inside this nuclear structure, which underpins calculation of the ratios presented in Fig. 4P and R). (N=8-10 measurements, n=5 cells, 2 biological replicates).

Figure S5 related to Figure 5. Localization of HR factors at heterochromatic DSBs in the absence of HP1s (A) Quantification of HP1α, Hp1β or HP1γ colocalization with SatIII domains in U2OS cells expressing a gRNA specific for SatIII repeats with or without Cas9-specific DSB induction. **(B)** Quantification of HP1γ colocalization with SatIII repeats in U2OS cells expressing a gRNA specific for SatIII repeats with or without Cas9-specific DSB induction in cells in different stages of the cell cycle. **(C)** Efficiency of siHP1αβγ siRNAs in U2OS and NIH-3T3 cells revealed by Western blot analysis. **(D)** Western blot analysis of the endogenous HP1α and GFP-HP1α or EGFP-HP1α^{I165E} levels in WT NIH-3T3 cells or NIH-3T3 cells depleted of Hp1αβγ, before and after expression of

GFP-HP1α or EGFP-HP1α^{I165E}. **(E)** Quantification of RAD51 pattern at chromocenters on G2-synchronized NIH-3T3 cells expressing Cas9 + gRNA targeting major satellite repeats, depleted of Hp1αβγ and complemented with EGFP-HP1α^{I165E} or GFP-HP1α. **(F)** IF confocal analysis of WT MEFs (upper panel) or SUV3/9 dKO MEFs (middle and lower panels) expressing Cas9 + gRNA targeting major satellite repeats stained with DAPI and antibodies specific for γ-H2AX and RAD51. The lower panel represents a representative image of a cell where RAD51 is not recruited. Quantification of RAD51 recruitment **(G)** and pattern **(H)** in WT MEFs and SUV3/9 dKO MEFs expressing Cas9 + gRNA targeting major satellite repeats. Images are representative of 3 experiments with n=50 cells. **(I)** Quantification of BRCA1 pattern in U2OS cells expressing Cas9 + gRNA targeting SatIII repeats, depleted of HP1αβγ (siHP1αβγ) or transfected with a non-targeting control cells (siSCR) in G1/S (left) and S/G2 (right) phase of the cell cycle. **(J)** Two-colour confocal image of wt HeLa cells or HeLa cells depleted of HP1α expressing mKate2 and DBD-TRIM-EGFP, where a line scan (white arrow) in the region of interest was selected for pCF analysis, enlarged (bottom row). pCF6-8 analysis of mKate2 access into HeLa heterochromatin foci before (grey) versus after (green) HP1α depletion (N = 10 measurements across N>=5 cells and 1 biological replicate). Scale bars represent 5 µm. Data are the mean ± SD of 3 experiments with n = 50 cells. Statistical significance was determined by t-test (*p<0,05, **p<0,01, ***p<0,001).

Figure S6 related to Discussion. Model of the spatial activation of DNA repair pathways in DSBs occurring in mouse and human heterochromatin. (A) In mouse cells, chromosomes are acrocentric and cluster through their pericentromeric repeats to form chromocenters. In S/G2, DSBs induced at the chromocenters relocate to the periphery of the domain to be repaired by HR and recruit Rad51.

At the same time, HP1 dimers are formed at the periphery of the domain, leading to reduced accessibility to HR factors. **(B)** In human cells, sat III repeats located on chromosome 9 do not exert the same degree of clustering. When DSBs are induced at satIII domains, the breaks remain positionally stable, the Hp1 dimers are reduced and the domain becomes accessible to HR factors, such as Rad51.

Table S1 (related to STAR METHODS), Probes for FISH

Probes for FISH		
**Probe**	**Company (reference)**	**Sequence**
TelG-Alexa488 PNA	Panagene (F1008)	G-rich probe (repeats of TTAGGG)
Sat III PNA - Alexa488	Panagene	Biotin-O-TTCCATTCCATTCCATTCCA
Sat II	Sigma	Biotin-TCGAGTCCATTCGATGAT-Biotin
Human chromosome 9	Applied Spectral Imaging (FPRPR0166)	

Table S2 (Related to STAR METHODS), Oligos

**Target**	**Forward**	**Reverse**
GAPDH (RT-qPCR)	AACTTTGGCATTGTGGAAGG	ACACATTGGGGGTAGGAACA
UNC45 (RT-qPCR)	TTTCCTGGCGAGCGGATCTA	TCAATCATGGGCACAGCCTT
Caf1 p150 siRNA oligos	AAGGAGAAGGCGGAGAAGCAG[dT][dT]	CUGGUUCUCCGCCUUCUCCUU[dT][dT] (antisense)

Table S3 (related to STAR METHODS), gRNA sequences

Guide RNAs used	***Sequence/Source***
g347 (sat III #1)	AATGGAATCAACACGAG
g349 (sat III #2)	TGGAATCAACCCGAGTAC
Ma-sat#3	Tsouroula et al. 2016
g501 (sat II)	CCAGTGTGAGCATCATCGAA
